# Supplementary material for: Zscan4 Is Activated after Telomere Shortening in Mouse Embryonic Stem Cells
Source: Stem Cell Reports. 2016 Mar 17;6(4):483–95. doi: 10.1016/j.stemcr.2016.02.010 (PMC4834046; doi:10.1016/j.stemcr.2016.02.010)
Supplement: Document S1. Supplemental Experimental Procedures and Figures S1–S7 [file mmc1.pdf]

Stem Cell Reports, Volume *6*

Supplemental Information

Zscan4 Is Activated after Telomere Shortening in Mouse Embryonic Stem Cells

Yoko Nakai-Futatsugi and Hitoshi Niwa

Supplemental Figures:

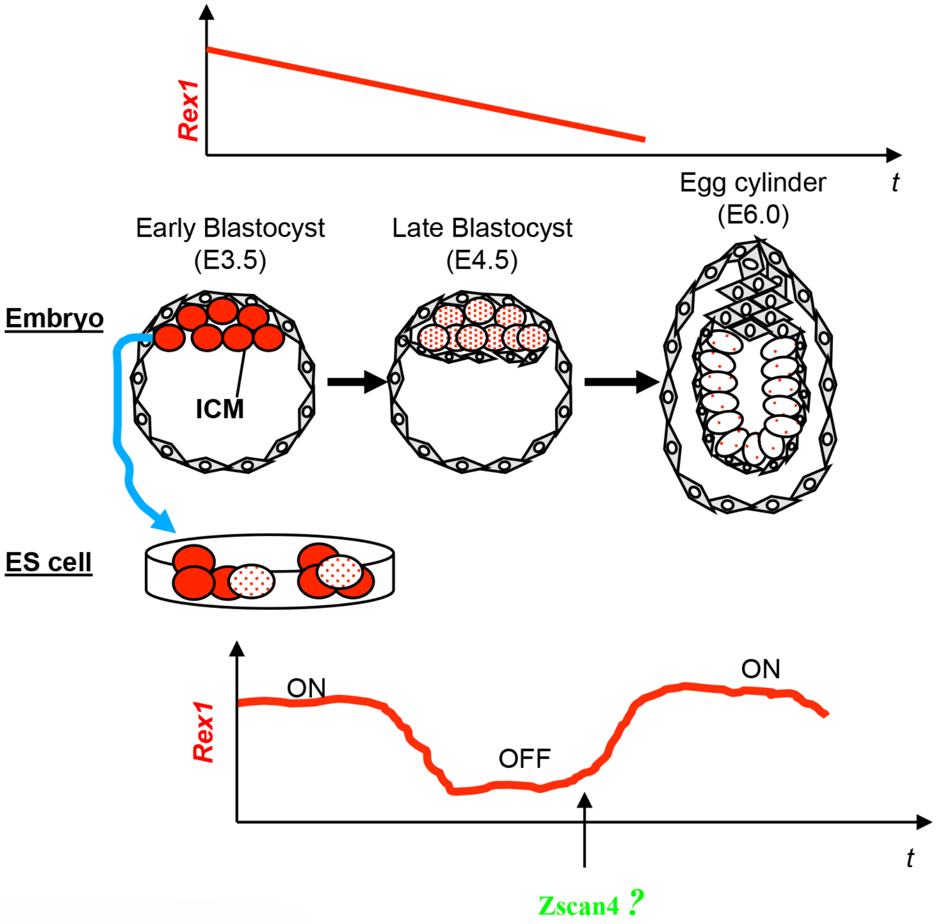

Figure S1, Related to Figure 2   Conceptual figure of the hypothesis

In vivo, the pluripotency-indicator *Rex1* is expressed only for 24 hours in the ICM during development, while *in vitro*, it is repeatedly up-regulated in mouse ESCs that derived from the ICM. To find the mechanism for this unnatural up-regulation, we will seek the possibility whether the "rejuvenation-factor" *Zscan4* plays the role.

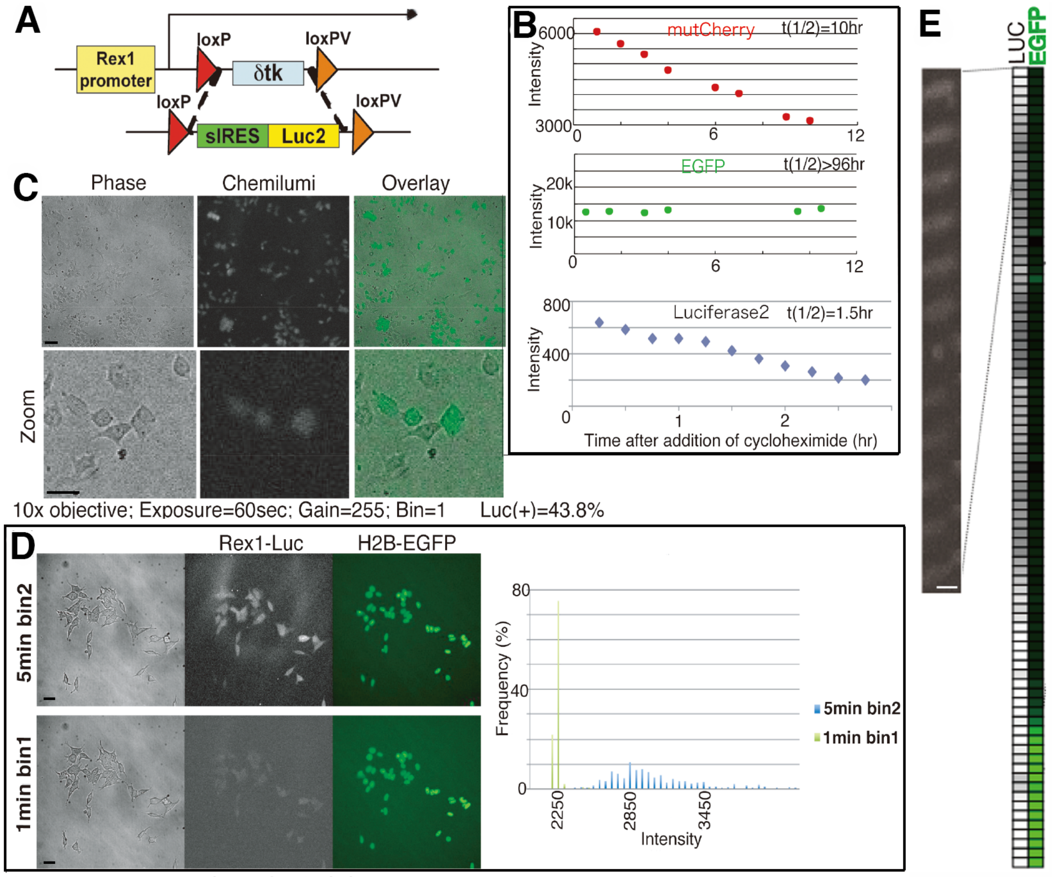

Figure S2, Related to Figure 2 Monitoring the *Rex1*-promoter activity

A Design of the exchange vector for *Rex1p*-Luc2. *Luciferase2* with "super-*IRES*" (Tsakiridis et al., 2009) fused on the 5' was flanked by *loxP* and a mutant *loxPV*, which was inserted by the cassette exchange system (Masui et al., 2005) into the downstream of the *Rex1* promoter of one of the alleles. **B** Half-lives of Luciferase2, EGFP, and muted mCherry were measured by the intensities of the images taken under the microscope following cycloheximide treatment. The half-life of Luciferase2 was as short as 1.5 hours while EGFP was very stable and even the fluorescent with the shortest half-life we were able to make, by adding mutated PEST domain (Li et al., 1998) to the N-terminal of monomeric Cherry, still had a much longer half-life. **C** Luciferase2, with its short half-life, allows monitoring of the kinetics of *Rex1*. *Rex1*-Luc showed 43% of Rex1-positive cells in culture, which was less than half compared to our previous study using *Rex1*-EGFP that showed 90% of *Rex1*-positive population (Toyooka et al., 2008). **D** Detection level of chemiluminescence depends on the exposure time. Exposure of 5 minutes gave clearer image (*left panel*) and broader distribution of intensity (*right panel*). This condition was used for all the experiments. **E** The intensity of the chemiluminescence (represents *Rex1* activity) and the intensity of GFP was converted to gray-scale and green-scale, respectively, using a handmade program written in Microsoft VBA. The actual image of the cell with slight down-regulation of *Rex1* is shown together with the gray-scale. Bars = 50μm (***C*** *upper*), 20μm (***C*** *lower* and ***D***) and 7.5μm (***E***).

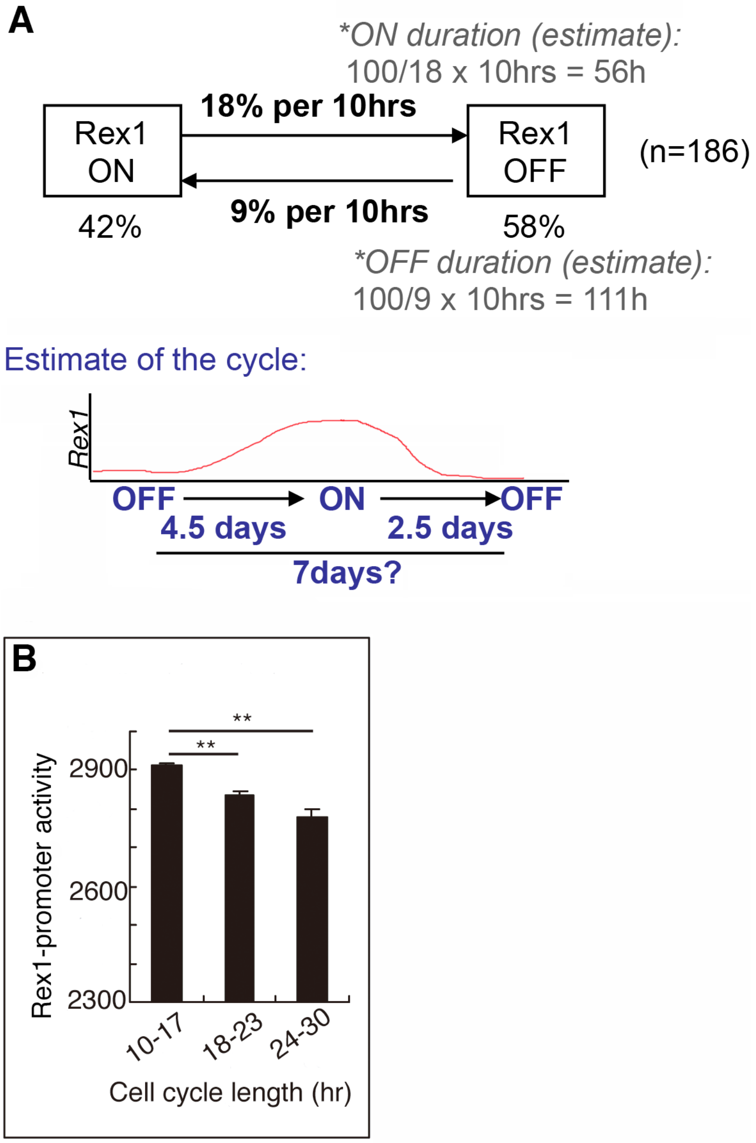

Figure S3, Related to Figure 2 ***Rex1*** **showed different kinetics from** ***Zscan4***

A Estimating the cycle length of the *Rex1*-fluctuation. By using the chemiluminescence Luciferase 2, the reversible up- and down-regulation of *Rex1* as previously predicted (Toyooka et al., 2008) was clearly visualized in the lineage tree (Figure 2C). However the cycle of the *Rex1*-fluctuation seemed very long, and it was not practical to measure the fluctuation by tracing the up and down repeat of one cell during the observation. So instead, many ESCs expressing *Rex1*-Luciferase were monitored for 10 hours under our live imaging system, and the number of the cells that turned from on to off and vice versa within 10 hours was used for the estimate. This rough estimate tells turning off seems to be easier than turning on, which is consistent with previous work showing that in the fluctuating wave of pluripotency, down-regulation of pluripotency-associated genes is faster than the up-regulation (Hayashi et al., 2008). In this estimate, we see the cycle of *Rex1*-fluctuation is not in the range of minutes or hours but of days or maybe of weeks. **B** *Rex1* promoter activity represented by *Rex1*-Luciferase intensity in the lineage tree was analyzed against the cell cycle length (n = 391 cells from 8 lineages from 2 experiments). At longer cell cycle, *Rex1* showed lower promoter activity. Bar graphs represent average with error bars of standard error. For statistical analysis, Student's T-test was used. **: $p < 0.005$.

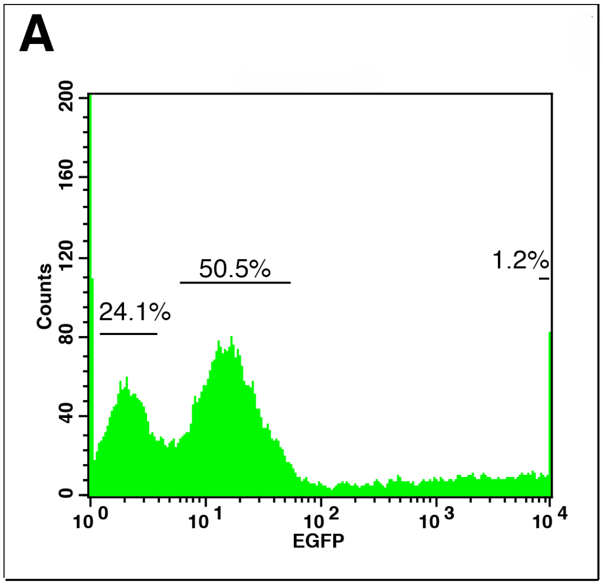

hours	GFPbk	GFP	log(GFP/bk)
50	7858.15	8114.24	0.013928
51	7885.28	8096.95	0.011504
52	7877.8	8161.81	0.015382
53	7884.08	8183.03	0.016163
54	7910.2	8165.38	0.013789
55	7923.36	8183.73	0.014042
56	7945.79	8214.46	0.014442

hours	GFPbk	GFP	log(GFP/bk)
69	7978.79	8696.33	0.037399
70	8010.56	9047.48	0.052865
71	7985.03	9710.52	0.084966
72	8012.77	10315.7	0.109716
73	8040.92	10706.3	0.124334
74	8037.16	11333.6	0.149265
75	8054.04	11935.9	0.170841

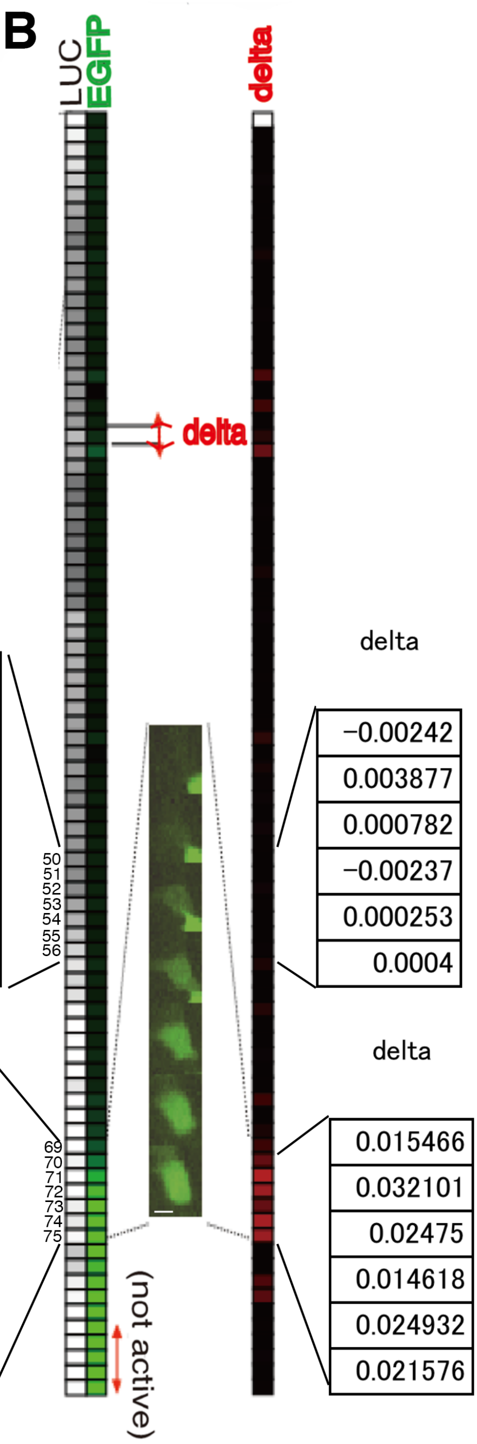

delta
−0.00242
0.003877
0.000782
−0.00237
0.000253
0.0004

delta
0.015466
0.032101
0.02475
0.014618
0.024932
0.021576

Figure S4, Related to Figures 2 and 3 **Detection of the *Zscan4*-pormoter activity by the Gal4-*UAS*-EGFP system**

A FACS analysis of the ESCs transfected with *Zscan4p*-Gal4 and *UAS*-EGFP vectors. There was 1.2% of the population with hyper-active *Zscan4* and notably 50.5% of weak positive population that was not detected in previous studies. **B** As the transactivator system amplified the GFP-intensity to a wide range, logarithm of the GFP-intensity subtracted by the logarithm of the background intensity was used to calculate the *delta* signal. The intention of using logarithm of the intensity was to fit the wide range of GFP-intensity into the 256-intensity-scale of the lineage tree, and also to adjust the effect of the fluctuation of the detection system (*i.e.* noise) that was higher at higher intensities. While positive *delta* indicates the promoter activity of *Zscan4*, considering the long half-life of EGFP, negative *delta* should indicate the degradation of the EGFP-protein and/or fluctuation of the detection system. Thus only the positive *delta* was used for the analysis in Figure 4. Bar = 7.5μm.

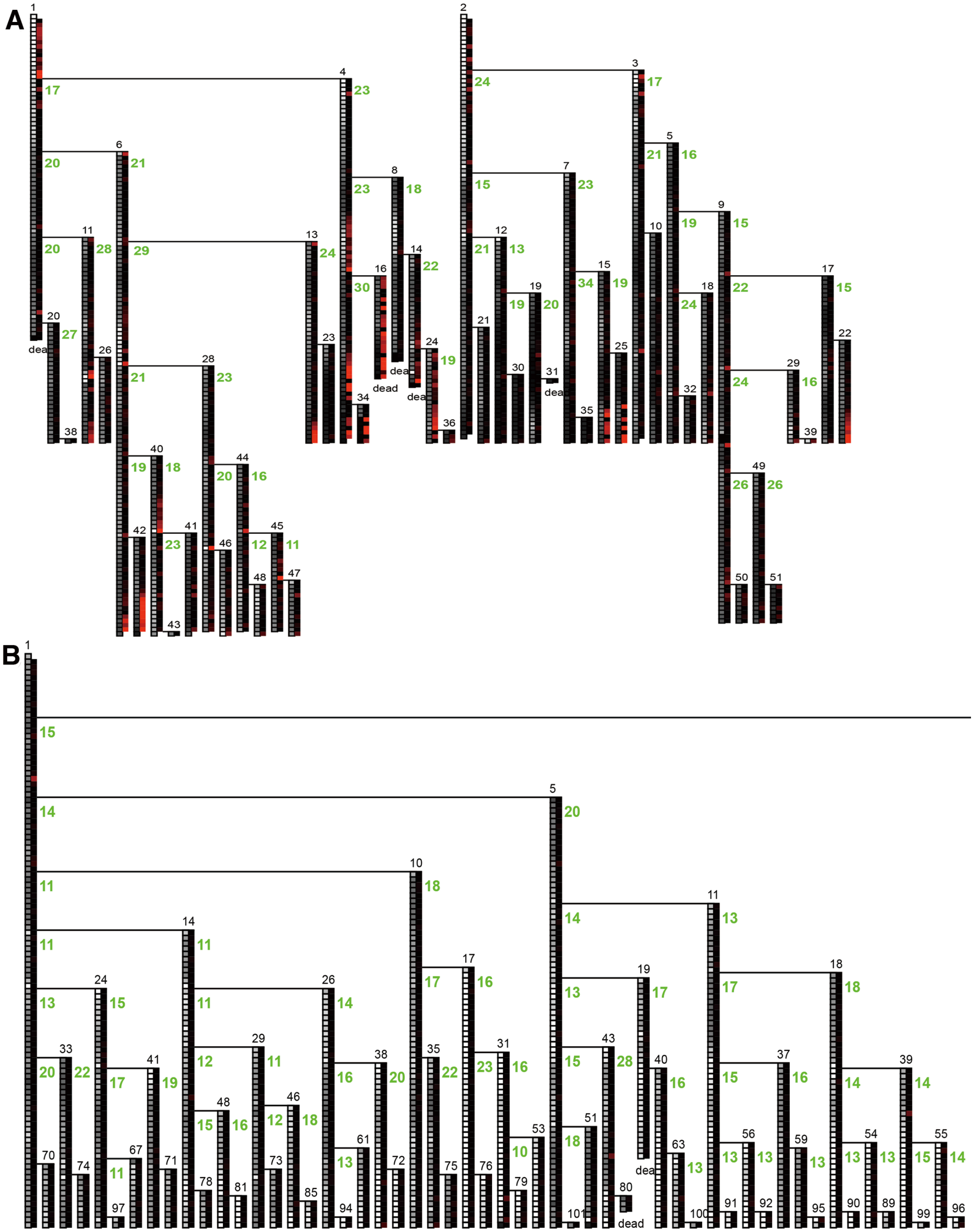

Figure S5, Related to Figure 3 **Two more examples of the lineage trees with *Rex1*-Luciferase and *delta* of *Zscan4*-Gal4-*UAS*-EGFP**

As shown in ***B***, *Zscan4* was less active when the cell cycle was stable.

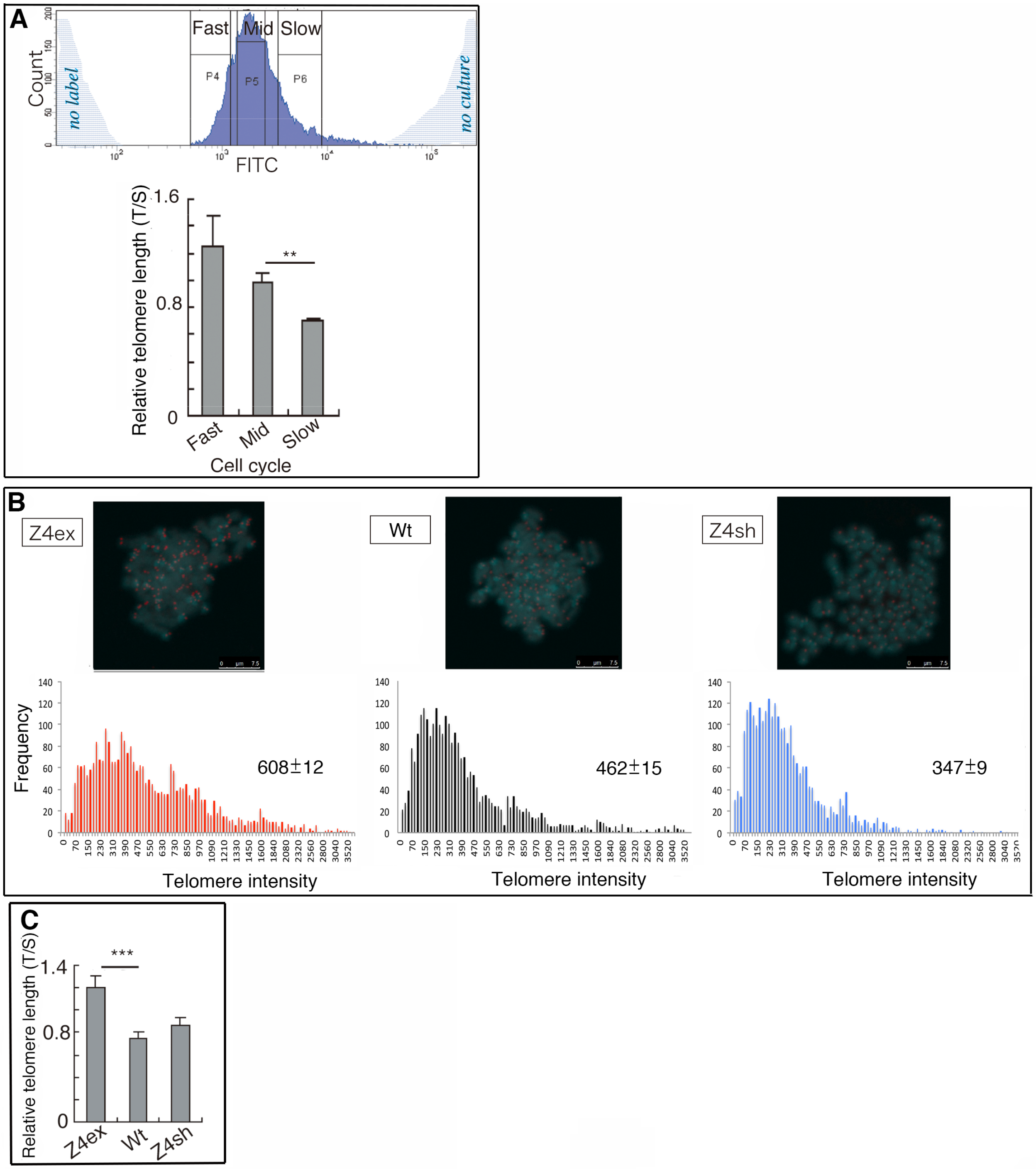

Figure S6, Related to Figures 5 and 6 **Telomere measurement by qPCR and qFISH**

A ESCs labeled with CFSE-dye followed by culture of 48 hours were sorted by FACS into three groups according to the dilution of the dye, which gives cohorts of fast, medium or slow cell cycle (*upper panel*). Telomere length of each cohort was measured by qPCR (n≥3 technical replicates) (*lower panel*). Telomeres were significantly short in the cells with longer cell cycles. **B** Telomere lengths of Z4ex-, wild type- or Z4sh- ESCs measured by qFISH (Bars = 7.5μm; 20 cells each from 2 technical replicates; n=2865, 2803 and 2681 telomeres, respectively). Average telomere intensities ± standard deviations are indicated. **C** Telomere lengths of Z4ex-, wild type- or Z4sh- ESCs measured by qPCR (n≥3 biological replicates) . Unlike qFISH (***B***) and flow-FISH (Figure 6A), the difference between wild-type and Z4-sh-ESCs was not detected, which could be due to the less accuracy of this method for telomere measurement (Gutierrez-Rodrigues et al., 2014). Bar graph represents average with error bars of standard deviation. For statistical analysis, Student's T-test was used. **: $p < 0.005$, ***: $p < 0.001$.

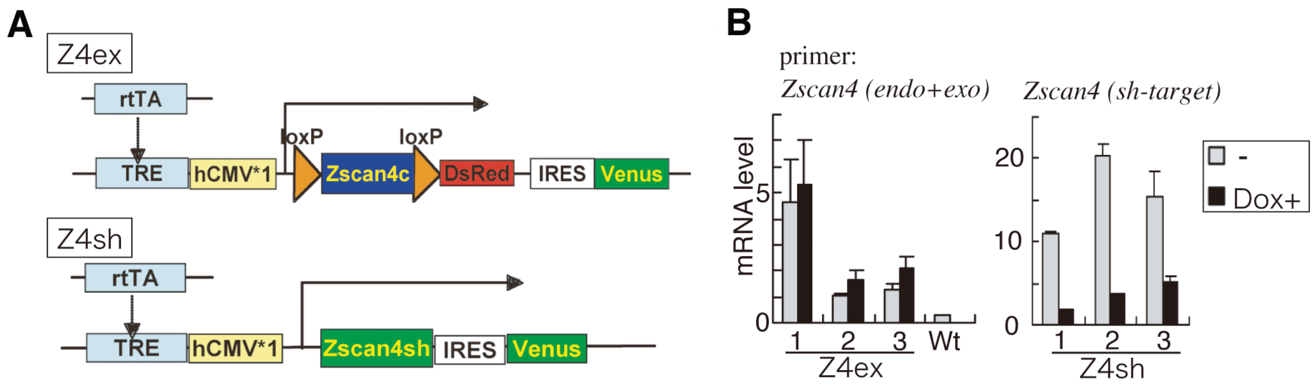

Figure S7, Related to Figure 6 Generation of the Z4ex- and Z4sh- ESCs

A Designs of the *Zscan4c* expression vector in which *Zscan4c* was coded downstream of the Tet-responsible element with a minimal CMV promoter (*upper*) and the Zscan4c knockdown vector in which the same shRNA as previously identified (Zalzman et.al., 2010) was used downstream of the Tet-responsible element with a minimal CMV promoter (*lower*). **B** Tet-inducible expression and knockdown were confirmed by qPCR analysis in Z4ex- and Z4sh- ESCs, using primer pairs that recognize both endogenous and exogenous *Zscan4c* (*endo+exo*) and the target sequence of the *Zscan4c*-shRNA (*sh-target*), respectively. Black bars indicate the Tet-system was ON by the addition of doxycycline (Dox). Bar graph represents average with error bars of standard deviation (n=3 technical replicates).

Note1: The vector design of Z4ex was made to avoid undesired level of over-expression that might kill the cells (Hirata et al., 2012), by flanking *Zscan4c* ORF by loxP so that it could be cleaved by the induction of Cre recombinase after transient expression of *Zscan4c* (Figure S6A). However, unexpectedly, there seemed to be a “leak” in the Tet-system and clones with optimal level of *Zscan4c* that was constitutively expressed even without doxycycline (Dox) were dominantly selected (Figure S6B, *Z4ex* vs *Wt*), which might be a similar case as reported previously (Amano et al., 2013). Driving the Tet-system by the addition of Dox (1 μg/ml) further increased the expression of *Zscan4c* (Figure S6B *Z4ex, Black bars*) but not to a toxic level, as seen by good proliferation and normal morphologies of these clones even in the presence of Dox. Thus to rule out ambiguous “leak” of the Tet-system, we used these clones only in the presence of Dox (i.e. the condition represented by the *Black bars* in Figure S6B *Z4ex*). For controls, instead of Z4ex-ESCs without Dox, wild type ESCs were used. Considering similar leak in Z4sh-ESCs, the Z4sh-ESC lines were used only in the presence of Dox as well (i.e. the condition represented by the *Black bars* in Figure S6B *Z4sh*), with wild type ESCs for controls.

Note2: The knockdown efficiency shown in ***B*** was 83%, 83% and 67% for clones Z4sh#1, #2 and #3, respectively. Clones #1 and #2 were used for the experiments. Although we used the same shRNA sequence as previous study (Zalzman et al., 2010), our knockdown was less effective than Zalzman et al. that showed 96% of reduction. Probably because while we used inducible shRNA, Zalzman et al. stably expressed the shRNA and over-expressed exogenous *Zscan4* during the establishment of the knockdown-clones, which should give more efficient knockdown by continuous expression of the shRNA.

Supplemental Experimental Procedures:

Telomere length measurement by quantitative fluorescent *in situ*-hybridization

Cells were treated with 100 ng/ml of colcemid (Nacalai Tesque, Kyoto, Japan) for 15-19 hours to enrich M-phase cells. Then the cells were trypsinized and incubated in 75mM of KCl at 37 ^{o}C for 20 min, fixed with methanol : acetic acid (3:1), spread on cover glass and dried by sequential wash with 70%, 85% and 96% ethanol. Then the cell-spread was incubated with 30 nM of TelG-Cy3 (Panagene) at 80 ^{o}C for 5 min followed by 2 hours at room temperature. Then the sample was washed sequentially with 70% formamid at room temperature, PBS with 0.1% Tween-20 at 57 ^{o}C and 2xSSC buffer with 0.1% Tween-20 at room temperature. The DNA were counter-stained with Hoechst33342 (Invitrogen).

Images were taken by SP8-confocal microscope (Leica) and the intensities were analyzed using MetaMorph imaging software. To create regions of interest, intensity threshold was set at 5/255 and objects with area size from 10 to 200 pixels were measured.

Telomere length measurement by quantitative PCR

Telomere lengths were measured by qPCR following the method previously described (Callicott and Womack, 2006). Ten ng of genomic DNA was used for 10 ml of reaction mixture of THUNDERBIRD SYBR qPCR Mix. Each reaction was made in triplets. A single copy gene *36B4* was used as an inner control. The telomere signal was normalized to *36B4* to generate a telomere-to-single copy gene (T/S) ratio indicative of relative telomere length. Sequences of primer pairs are as follows:

Telomere (Callicott and Womack, 2006)

forward 5'-CGG TTT GTT TGG GTT TGG GTT TGG GTT TGG GTT TGG GTT-3';

reverse 5'-GGC TTG CCT TAC CCT TAC CCT TAC CCT TAC CCT TAC CCT -3',

36B4 (Callicott and Womack, 2006)

forward 5'-ACT GGT CTA GGA CCC GAG AAG -3';

reverse 5'-TCA ATG GTG CCT CTG GAG ATT -3'.

Supplemental Reference:

Li, X., Zhao, X., Fang, Y., Jiang, X., Duong, T., Fan, C., Huang, C.C., Kain, S.R. (1998). Generation of destabilized green fluorescent protein as a transcription reporter. *J. Biol. Chem.* **273**. 34970-34975.

Supplemental Material:

Source code S1, Related to Figures 2 and 3 **Source code of the program to draw the lineage tree**

This material is provided in a format of a Microsoft Excel sheet (Excel 2003). It includes the hand made program to draw the lineage trees together with the actual input data of Figures 2B and 3C. The source code of the program is embedded as a VBA macro, which can be read and edited by the Microsoft Excel Menu [Tools]-[Macro]-[Visual Basic Editor].
