# Supplementary material for: Zscan4 Is Activated after Telomere Shortening in Mouse Embryonic Stem Cells
Source: Stem Cell Reports. 2016 Mar 17;6(4):483–95. doi: 10.1016/j.stemcr.2016.02.010 (PMC4834046; doi:10.1016/j.stemcr.2016.02.010)
Supplement: Document S2. Article plus Supplemental Information [file mmc3.pdf]

Stem Cell Reports
Article
OPEN ACCESS

Zscan4 Is Activated after Telomere Shortening in Mouse Embryonic Stem Cells

Yoko Nakai-Futatsugi[1,3,*] and Hitoshi Niwa[1,2,4,*]
[1]Laboratory for Pluripotent Stem Cell Studies, RIKEN Center for Developmental Biology, 2-2-3 Minatojima-minamimachi, Chuo-ku, Kobe 650-0047, Japan
[2]Japan Science and Technology Agency, CREST, Sanbancho, Chiyoda-ku, Tokyo 102-0075, Japan
[3]Present address: Center for iPS Cell Research and Application, Kyoto University, 53 Kawahara-cho, Shogoin, Sakyo-ku, Kyoto 606-8507, Japan
[4]Present address: Department of Pluripotent Stem Cell Biology, Institute of Molecular Embryology and Genetics, Kumamoto University, 2-2-1 Honjo, Chuo-ku, Kumamoto 860-0811, Japan
*Correspondence: y.futatsugi@cira.kyoto-u.ac.jp (Y.N.-F.), niwa@kumamoto-u.ac.jp (H.N.)
http://dx.doi.org/10.1016/j.stemcr.2016.02.010
This is an open access article under the CC BY license (http://creativecommons.org/licenses/by/4.0/).

SUMMARY

ZSCAN4 is a DNA-binding protein that functions for telomere elongation and genomic stability. In vivo, it is specifically expressed at the two-cell stage during mouse development. In vitro, it is transiently expressed in mouse embryonic stem cells (ESCs), only in 5% of the population at one time. Here we attempted to elucidate when, under what circumstances, *Zscan4* is activated in ESCs. Using live cell imaging, we monitored the activity of *Zscan4* together with the pluripotency marker *Rex1*. The lengths of the cell cycles in ESCs were diverse. Longer cell cycles were accompanied by shorter telomeres and higher activation of *Zscan4*. Since activation of *Zscan4* is involved in telomere elongation, we speculate that the extended cell cycles accompanied by *Zscan4* activation reflect the time for telomere recovery. *Rex1* and *Zscan4* did not show any correlation. Taken together, we propose that *Zscan4* is activated to recover shortened telomeres during extended cell cycles, irrespective of the pluripotent status.

INTRODUCTION

Zinc finger and SCAN domain containing 4 (ZSCAN4) is a DNA-binding protein that is specifically expressed in two-cell stage embryos during mouse development (Falco et al., 2007). In vitro, interestingly, *Zscan4* is transiently expressed in a minor population of embryonic stem cells (ESCs) at one time (Carter et al., 2008) but is eventually expressed in all (Zalzman et al., 2010). It functions for telomere elongation and genomic stability (Zalzman et al., 2010) and thus is considered as a rejuvenation factor.

ESCs are a heterogeneous population. If cultured in conventional serum-containing medium supplemented with leukemia inhibitory factor (LIF), they remain undifferentiated but closer studies show they are actually a mixture of cells with higher and lower potential of differentiation (reviewed in Nakai-Futatsugi and Niwa, 2013). Recently even a minor population of two-cell-stage-like ESCs that are not only pluripotent but also capable of differentiating into extra-embryonic lineages was found in the heterogeneous ESC population (Macfarlan et al., 2012). The heterogeneity of ESCs is accompanied by fluctuation of the expression of pluripotency-associated genes such as *Rex1* (also known as *Zfp42*) (Toyooka et al., 2008), *Nanog* (Chambers et al., 2007; Singh et al., 2007), *Klf4* (Niwa et al., 2009), *Tbx3* (Niwa et al., 2009), *Stella* (Hayashi et al., 2008), and so on. However, among the pluripotency-associated genes, *Oct3/4* (also known as *Pou5f1*), whose expression does not fluctuate, is an exception. It is the master gene of pluripotency (Nichols et al., 1998). A constant expression level of *Oct3/4* is crucial for the maintenance of pluripotency, as a slight increase leads to differentiation into primitive endoderm and mesoderm while a slight decrease leads to differentiation into trophoectoderm (Niwa et al., 2000). The expression level of *Oct3/4* is maintained at a constant level downstream of a robust transcription factor network in mouse ESCs (Niwa et al., 2009). *Rex1*, although not essential for ESC self-renewal and pluripotency (Masui et al., 2008), decreases in its mRNA-expression level when the master gene *Oct3/4* is either hyper-expressed or hypo-expressed (Niwa et al., 2000). Thus we consider the promoter activity of *Rex1*, which is high only when the expression of *Oct3/4* is maintained at an optimal range, as a good indicator of pluripotency.

To elucidate whether the expression pattern of *Zscan4* has any correlation with ESC proliferation, we monitored *Zscan4* activity at single cell level. Also to see whether the rejuvenation factor *Zscan4* correlates with the fluctuating wave of ESC pluripotency (Figure S1), we monitored *Zscan4* and the pluripotency indicator *Rex1* simultaneously under live cell imaging. Unexpectedly, we did not see any correlation between the two factors. Instead, we found *Zscan4* is activated when the cell-cycle lengths become long, irrespective of the pluripotent status, presumably sensing shortened telomeres.

RESULTS

Cell-Cycle Length of Mouse ESCs Is Diverse

First we analyzed the proliferation profile of ESCs at the single cell level. ESCs were stably transfected with Fucci vector

CrossMark

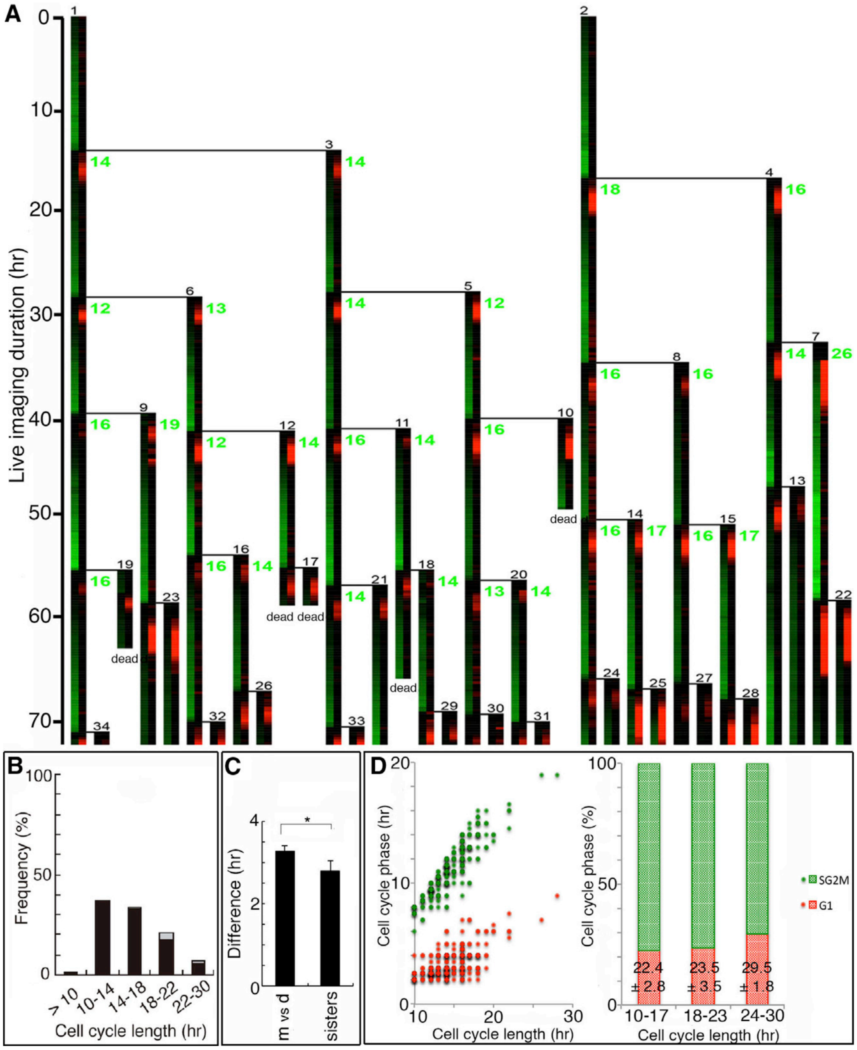

Figure 1. The Cell Cycle of ESCs Is Diverse

(A) Examples of lineage trees generated by live imaging of ESCs expressing Fucci vector. Each vertical line shows the fate of each cell plotted for every time point, where red and green indicate the G1 phase and the S/G2/M phase, respectively. Horizontal lines indicate cell division. Cells were sequentially numbered in the order they emerged (small black numbers). Timescale is on the left of the lineage tree. Green numbers indicate the cell-cycle length (hr).

(B) Histogram of the cell-cycle length of ESCs cultured in conventional media (n = 172 cell divisions in six lineages from two independent experiments). Black, the cells whose daughter cells divided within 30 hr; gray, the cells whose daughter cells died or did not divide within 30 hr.

(C) The cell-cycle lengths between mother and daughter, and between sisters were compared by the absolute value of the difference (n = 192 cell divisions in four lineages from two independent experiments). The difference between sisters was significantly small. m, mother; d, daughter. Bar graph represents the average with error bars of SE. Student's t test was used for statistical analysis. $*p < 0.05$.

(D) Quantification of the length of the G1 phase (red) and the S/G2/M phase (green) from the lineage trees as shown in (A) (n = 284 cell divisions in five lineages from two independent experiments).

(Sakaue-Sawano et al., 2008), which expresses fluorescence Kusabira orange at the G1 phase and fluorescence Azami green at the S/G2/M-phase. They were monitored under the microscope for up to 5 days in conventional medium that contains fetal calf serum (FCS) supplemented with leukemia inhibitory factor (LIF) (FCS/LIF medium). Images were taken every 15 min. After the images were taken, each cell was tracked manually and the data were converted into lineage trees using a handmade program (source code provided in Data S1). Figure 1A shows examples of the lineage trees, in which each vertical line shows the fate of each cell, plotted for every time point with the intensities of Kusabira orange and Azami green converted into 256 intensity scale of red and green, respectively. Horizontal lines indicate cell division. Cells were sequentially numbered in the order they emerged (small black numbers). The timescale is on the left of the lineage tree. Green numbers indicate the cell-cycle length (hr). Although previous studies have suggested the cell-cycle length of mouse ESCs should be around 10–14 hr (Pauklin et al., 2011), under our conditions, the length of the cell cycle was more diverse than expected; it varied from less than 10 hr to more than 20 hr (Figures 1A, 2C, and S5, green numbers; see also Figure 1B). Interestingly, the cell cycles of the sister cells were similar (Figures 1A, 2C, and S5, compare green numbers between sisters), probably because the cell components including the cell-cycle determinants were divided evenly between the daughter cells. When the difference in the cell-cycle length between mother and daughter, and between sisters were quantified, sister cells showed a significantly smaller difference (Figure 1C).

The G1 phase in ESCs is characteristically short. Typically the G1 phase occupies only 20% of the total cell-cycle length in ESCs, while in somatic cells it is more than 50% (White and Dalton, 2005). Our Fucci-based live imaging confirmed that cells at longer cell cycles still had the typical ESC-like

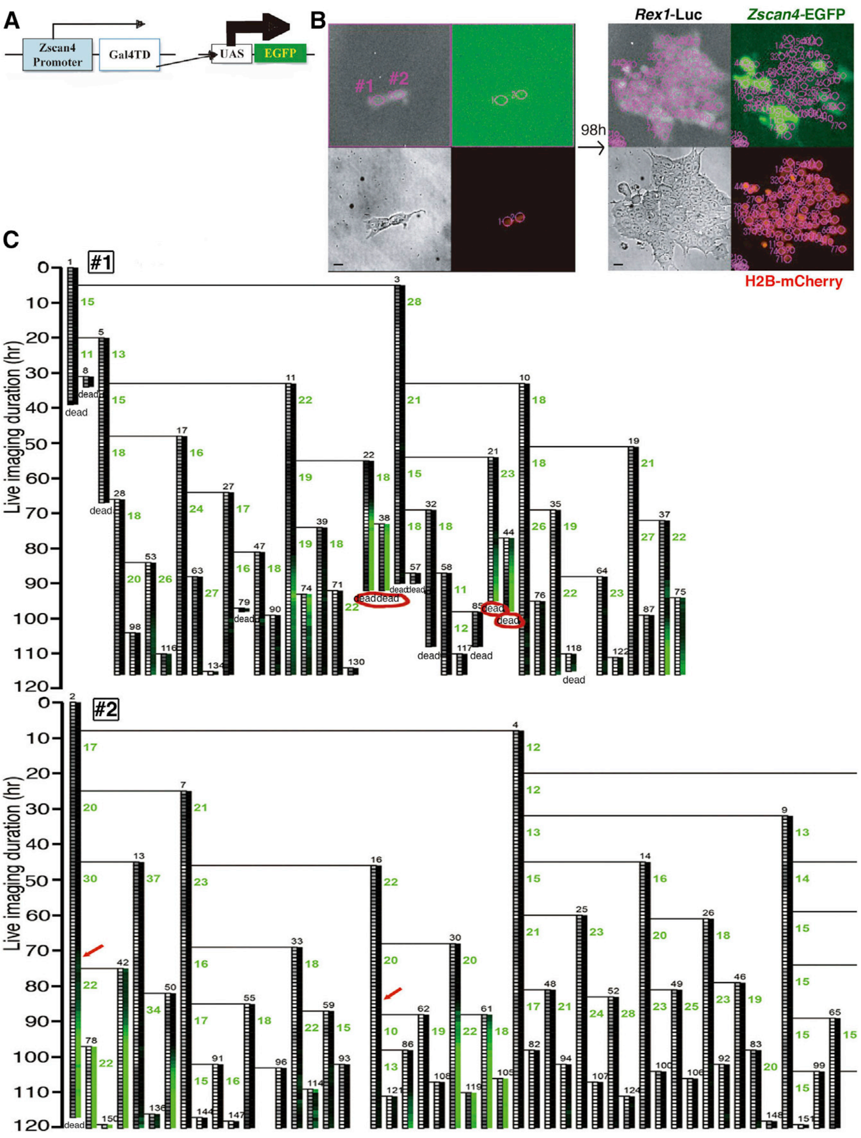

Figure 2. Live Imaging of *Zscan4* and *Rex1*

(A) Design of the *Zscan4* reporter transgene. Upon the activity of the *Zscan4* promoter, the *trans*-activator domain of GAL4 is expressed, which *trans*-activates the co-transfected *UAS* promoter with *EGFP* downstream. See also Figure S4A.

(B) Example of cell tracking starting from two cells (#1 and #2). mCherry fused to H2B was used as a nuclear marker. Images were captured every 60 min up to 120 hr. A region of the nucleus in each cell was drawn manually and intensities of Luciferase2 (for *Rex1*) and GFP (for *Zscan4*) were measured. Bars, 10 μm.

(C) Lineage trees generated from the cell tracking of cells #1 and #2 in (B). Green numbers indicate the cell-cycle length (hr). Intensities of Luciferase and GFP (described in Figures S2E and 3A, respectively) are shown by color scales. Input data and the program to draw the lineage trees are provided in Data S1. Red arrows indicate subtle GFP intensities, and red circles indicate cell death in GFP-strong-positive cells (both are discussed in Figure 3B). During culture, cells seemed to die randomly. However, from the lineage tree, we noticed that most of the dead cells derived from cell #1. This means that the death fate was already determined in the two apparently identical cells shown in (B) (#1 and #2). See also Figures S1–S3 *for Rex1*.

ratio of the G1 phase (Figure 1D, right panel). At the same time, this meant that not only the G1 phase but also the actual duration of the S/G2/M phase was elongated in ESCs when the cell cycles became longer (Figure 1D, left panel).

Monitoring *Zscan4* Promoter Activity

We monitored the promoter activities of *Zscan4* and the pluripotency marker *Rex1* under our live imaging system. For monitoring of *Rex1* promoter activity, chemiluminescence *Luciferase2* was knocked in downstream of the *Rex1* promoter (Figure S2A). We used Luciferase2, which has a short half-life, for quasi real-time imaging of *Rex1* promoter activity (Figure S2). With this probe, the fluctuation of *Rex1* promoter activity was clearly visualized (Figure S2E) and the cycle of fluctuation was estimated to be approximately 7 days (Figure S3A). *Rex1* promoter activity showed negative correlation with the cell-cycle length (Figure S3B; also discussed in Figure 7A).

For monitoring of *Zscan4* promoter activity, ESCs were stably transfected with a set of transgenes that express enhanced green fluorescent protein (EGFP) downstream of *Zscan4* promoter (Figure 2A). Images were taken every 1 hr. Figure 2B shows an example of the live imaging data starting from two cells (#1 and #2). Marks of manual tracking are shown by pink circles. Figure 2C shows the lineage trees of cells #1 and #2 in Figure 2B. Each vertical line shows the fate of each cell plotted for every time point with the intensities of *Rex1* Luciferaase2 and *Zscan4* EGFP converted into a 256 intensity scale of gray and green, respectively. As shown in Figure 2C, cells with strong GFP

intensities were prone to die (red circles), which was distinguished from autofluorescence of dying cells as it was not detected through an red fluorescent protein filter. This was consistent with our previous study using a *Zscan4*-mCherry reporter showing that cells with hyperactive *Zscan4* had higher incidence of cell death (Fujii et al., 2015). With our previous version of the *Zscan4* probe that coded fluorescence mCherry directly downstream of the *Zscan4* promoter, most likely only the hyperactive *Zscan4* that led to cell death was detectable. Thus, in order to elucidate the function of *Zscan4*, we thought that detecting much weaker *Zscan4* activity might be required. *trans*-Activator systems are generally used for signal amplification (Iyer et al., 2001) and our preliminary study showed *trans*-activation by Gal4-*UAS* amplifies the signal by 10–100 times (H.N., unpublished data). So we applied the Gal4-*UAS*-*trans*-activator system to amplify small *Zscan4* signals into large expression levels of the EGFP reporter (Figure 2A). The signal amplification by the Gal4-*UAS*-*trans*-activator system was indeed efficient as the population with a weak *Zscan4*-GFP signal that was not detected in previous studies emerged by fluorescence-activated cell sorting (FACS) analysis (Figure S4A). This enabled the visualization of very weak signals as shown in Figure 2C (red arrows).

While Luciferase2 was efficient for monitoring the kinetics of the *Rex1* promoter activity due to its short half-life (Figure S2), the intensity of EGFP itself does not directly indicate promoter activity of *Zscan4* because the half-life of EGFP is rather long (Figure S2B) so may not degrade even after the promoter is "off" (Figure 3A, "not active"). To interpret the kinetics of the *Zscan4* promoter activity from the EGFP intensity, we used the increment of the EGFP signal during one time point (Figure 3A, delta; see also Figure S4B) as a variable that represents the promoter activity (Figure 3A, red scale). Figure 3B is the converted version of Figure 2C with the *delta* of EGFP plotted in red. The activities of *Zscan4* that had weaker GFP intensities are now elucidated (Figure 3B, red arrows).

We attempted to see whether *Zscan4* boosts *Rex1* expression or vice versa, but there was no correlation between the promoter activities of *Rex1* and *Zscan4* (Figure 3C).

Zscan4 Is Activated at Longer Cell Cycles

From the lineage trees (Figure 3B; see also Figure S5), we noticed that *Zscan4* tended to become active when the cell-cycle length became long. Thus we quantified the average of the *Zscan4* promoter activity (represented by the *delta* of the EGFP signal) during one cell cycle and plotted it against the length of that cell cycle (Figure 4A). As a result, indeed there was a significant upregulation of *Zscan4* promoter activity at longer cell cycles (around 20 hr) (Figure 4A). Accordingly, an ESC that proliferated with constantly short cell-cycle lengths did not show strong upregulation of *Zscan4* (Figure S5B). We did the same analysis in mouse ESCs cultured in 2i/LIF medium (Ying et al., 2008), which consists of inhibitors for Map kinase kinase (MEK) and glycogen synthase kinase 3 beta (GSK3β) (2i) supplemented with LIF. Under this condition, naive pluripotency is maintained in mouse ESCs without fluctuation of the pluripotency-associated genes. The cell-cycle lengths were diverse as in conventional FCS/LIF culture, although 1.5–2 times longer in the presence of the two inhibitors (2i). Notably, activation of *Zscan4* was still accompanied by longer cell cycles even in 2i/LIF culture conditions (Figure 4B).

A previous study has shown that ZSCAN4 directly elongates the telomere by homologous recombination following the recruitment of SPO11 (Zalzman et al., 2010). Also it has been shown that *Zscan4* is activated in response to artificial telomere shortening by the depletion of telomerase (Huang et al., 2011). Thus, we thought that the expression of *Zscan4* at longer cell cycles may indicate activation of *Zscan4* in response to telomere shortening, which extends the cell-cycle length during repair of the telomere, presumably at the G2/M phase.

Zscan4 Activity Is Followed by Shortening of the Cell-Cycle Length in the Next Generation

If *Zscan4* is activated for repair by sensing shortened telomeres, the upregulation of *Zscan4* should be followed by shortening of the cell cycle after the recovery of the telomere. Thus we analyzed the correlation between the *Zscan4* promoter activity and the change in the cell-cycle length in the next generation. For this we quantified the total activity of *Zscan4* during one cell cycle, and plotted it against the difference between the length of that cell cycle and the next cell cycle (which becomes negative when the cell cycle shortens). As shown in Figure 4C, when the cell cycle was shortened, it was preceded by a significantly high activity of *Zscan4*. The same tendency was also shown in 2i/LIF culture conditions (Figure 4D).

Telomere Shortening Accompanies Cell-Cycle Elongation

As shown in Figure 2C (see also Figures 1A and S5), the cell-cycle length of ESCs fluctuates. It is noteworthy that the cell cycles not only elongate but also shrink. As *Zscan4* was active at longer cell cycles (Figures 4A and 4B) and the activation of *Zscan4* led to shrinkage of the length of the next cell cycle (Figures 4C and 4D), next we attempted to measure the telomere length in cohorts of ESCs that had short, medium, or longer cell cycles. ESCs that were labeled with carboxyfluorescein succinimidyl ester (CFSE) dye were cultured for 48 hr and collected according to the dilution of the dye (Figure 5, upper panel). We speculate that when

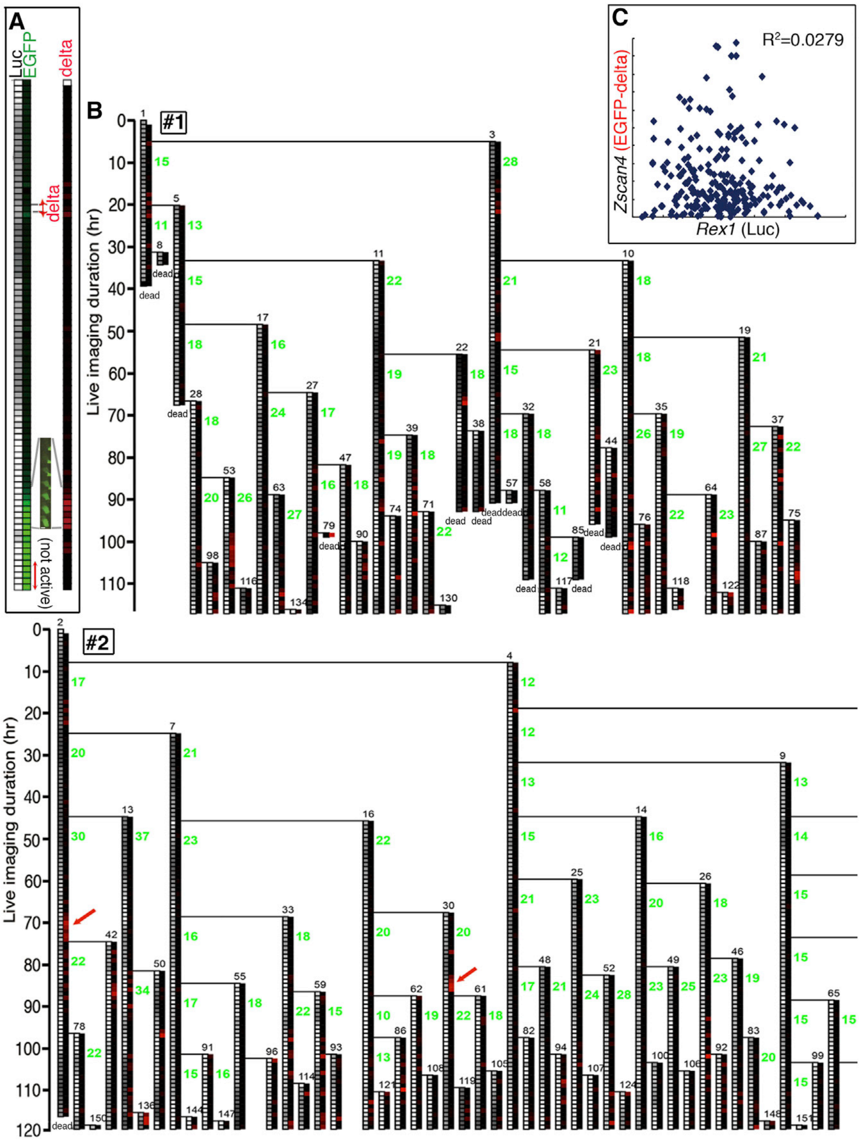

Figure 3. Monitoring *Zscan4* Promoter Activity

(A) The intensity of the chemiluminescence (represents *Rex1* activity) and the logarithm of GFP intensity were converted to a gray scale and green scale, respectively, using a handmade program written in Microsoft VBA (Data S1). Due to the long half-life of EGFP (Figure S2B), GFP positive does not necessarily mean *Zscan4* active. To evaluate *Zscan4* promoter activities, the GFP intensities were converted to the increment of the intensity (*delta* indicated in the green scale; i.e., GFP(t + 1) − GFP(t)) as shown in the red scale. The actual image of the cell that showed upregulation of *Zscan4* is shown together with the green and red scales. Bar, 7.5 μm. See also Figure S4B.

(B) The lineage trees in Figure 2B were converted to *delta* signal. Subtle *Zscan4* activity emerged (red arrows). See also Figure S5.

(C) *Rex1* and *Zscan4* promoter activities obtained from the intensities of Luciferase and GFP, respectively, were calculated as the average per hour within each cell cycle (n = 391 cell divisions in eight lineages from two independent experiments). There was no correlation ($R^2 = 0.0279$).

the telomere becomes short, time for telomere recovery presumably at the G2/M phase is required, and as a consequence the cell-cycle length becomes long. A standard method for telomere length measurement that applies quantitative fluorescent in situ hybridization (qFISH) (Lansdorp et al., 1996) only allows measurement of cells at the M phase, which may count only the cells after recovery of the telomeres, especially for longer cell cycles. So we applied a method using flow cytometry (flow-FISH) (Rufer et al., 1998; Baerlocher et al., 2006) and a method using quantitative PCR (qPCR) for telomere measurement (Callicott and Womack, 2006), which are both advantageous as a large number of unbiased cells, not only at the M phase, can be measured. Of course telomere shortening is not the only cause of cell-cycle elongation (this is discussed later in Figure 7). Thus ESCs that had longer cell cycles during the last 48 hr should be a mixture of several conditions. Nevertheless, the cohort of ESCs that had longer cell cycles had significantly short telomeres (Figure 5, see also Figure S6A). This again supports the idea that the activation of *Zscan4* at longer cell cycles might be a consequence of sensing telomere shortening.

ESCs with Constitutive Expression of *Zscan4* Have Stable Cell-Cycle Length

We generated ESC lines that stably express exogenous *Zscan4c* (Z4ex-ESC) (Figure S7, Z4ex). When the telomere length was measured by flow-FISH (Figure 6A), qFISH (Figure S6B), and qPCR (Figure S6C) Z4ex-ESCs had longer telomeres, consistent with the pioneer study (Zalzman et al.,

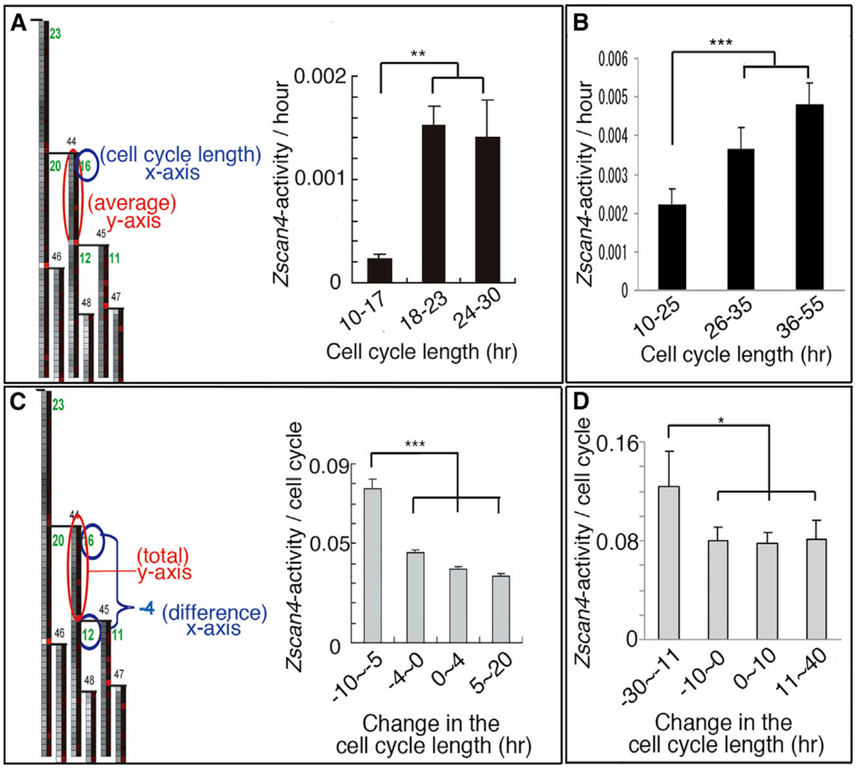

Figure 4. *Zscan4* Activity Correlates with the Cell-Cycle Length

(A and B) *Zscan4* is activated when the cell-cycle length is long. *Zscan4* promoter activity represented by the *delta* of GFP intensity in the lineage trees was analyzed against the cell-cycle length in FCS/LIF culture conditions (A; n = 386 cell divisions in eight lineages from two independent experiments) or in 2i/LIF culture conditions (B; n = 283 cell divisions in four lineages from two independent experiments). *Zscan4* activity became significantly high when the cell-cycle length became long.

(C and D) Expression of *Zscan4* leads to shortening of the cell cycle at the next cell division. Total *Zscan4* activity during a given cell cycle was analyzed against the difference between the lengths of that cell cycle and the next cell cycle in FCS/LIF culture conditions (C; n = 276 cell divisions in eight lineages from two independent experiments) or in 2i/LIF culture conditions (D; n = 255 cell divisions in four lineages from two independent experiments). Negative x axis means the cell cycle shortened. Bar graph represents the average with error bars of the SD. Student's t test was used for statistical analysis. *$p < 0.05$, **$p < 0.005$, ***$p < 0.001$.

2010), demonstrating the function of *Zscan4* in telomere elongation. With these Z4ex-ESCs, we analyzed whether the expression of *Zscan4* affects the cell-cycle length. Examples of the lineage trees of Z4ex-ESCs are shown in Figure 6B. In Z4ex-ESCs, the majority of the cell-cycle length remained within 15 hr (Figure 6B; see also Figure 6D, Z4ex), which was relatively short compared with wild-type ESCs (Figure 1B). Probably in Z4ex-ESCs with sufficient telomere length (Figures 6A, S6B, and S6C), time for telomere recovery is not required, and thus the cell-cycle lengths remain short.

Zscan4 Is Required for Survival of ESCs with Longer Cell Cycles

As the activation of *Zscan4* was followed by shortening of the cell-cycle length (Figures 4C and 4D), next we analyzed whether the downregulation of *Zscan4* affects the recovery from elongated cell cycle. We knocked down *Zscan4* by shRNA (Z4sh-ESC) (Figure S7, Z4sh). Although we used the same shRNA sequence as in a previous study (Zalzman et al., 2010), our knockdown was less effective (83% and 96% of reduction, respectively). This was probably because, while we used inducible shRNA, Zalzman et al. (2010) stably expressed the shRNA and overexpressed exogenous *Zscan4* during the establishment of knockdown, which should give more efficient knockdown by continuous expression of shRNA (Figure S7). Thus unlike the *Zscan4* knocked down ESCs of Zalzman et al. (2010) that ceased to proliferate after eight passages, our Z4sh-ESCs slowly proliferated without crisis for at least 19 passages. Figure 6C shows examples of the lineage trees of Z4sh-ESCs. Z4sh-ESCs showed higher incidence of cell death (Figure 6C). The distribution of overall cell cycle length in Z4sh-ESCs did not alter much from wild-type ESCs (Figure 6D compared with Figure 1B). This suggests that the majority of the cells with presumably sufficient length of telomeres were not affected by the downregulation of *Zscan4*, which is reasonable considering the transient activation of *Zscan4* in ESCs. Our results so far suggest that *Zscan4* is activated by sensing shortened telomeres. Probably during the repair of the telomeres, the cell cycle lengthens followed by shortening of the cell cycle after recovery. If this is the case, the question is whether Z4sh-ESCs could survive after elongated cell cycles without sufficient activation of *Zscan4*. The gray portion of the histograms in Figure 6D indicates the number of the cells whose daughter cells could not proliferate. Z4sh-ESCs became less proliferative (died or did not divide for more than 30 hr) after longer cell cycles. For quantification, the ratio of the surviving cells (Figures 6D and

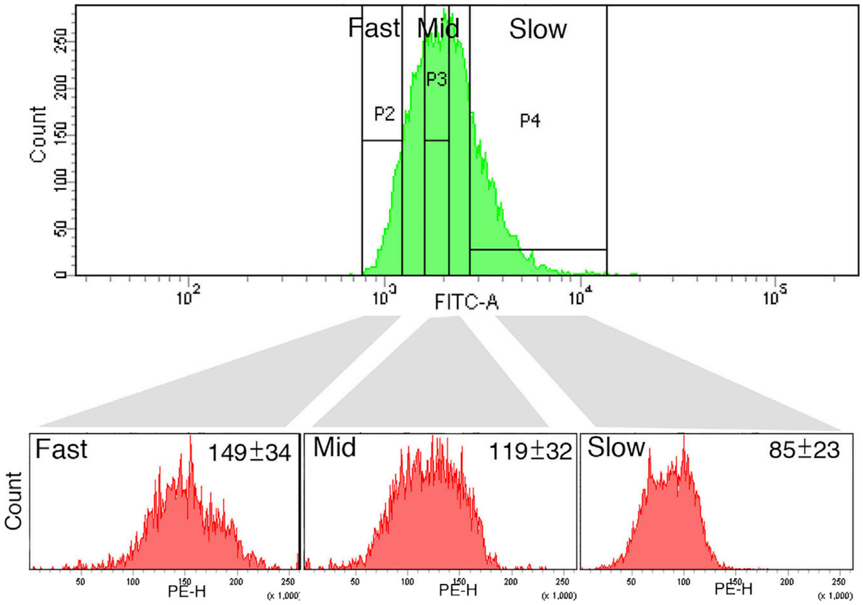

Figure 5. Telomere Shortening Accompanies Cell-Cycle Elongation

ESCs labeled with CFSE dye followed by culture for 48 hr were sorted by FACS into three groups according to the dilution of the dye, which gives cohorts of fast, medium, or slow cell cycles (upper panel). The telomere length of each cohort was measured by flow-FISH using a Cy3-conjugated telomere probe (lower panels). The average telomere intensity (×1,000) per cell ±SD is indicated. n > 5,000 cells. Two technical replicates showed the same tendency. Telomeres were shorter in the cells with longer cell cycles. See also Figure S6A.

1B, black portion versus total) was calculated (Figure 6E), which indicated that knockdown of *Zscan4* significantly reduced the number of the cells that could survive or recover from longer cell cycles. By qPCR, which is less sensitive for telomere measurement compared with flow-FISH or qFISH (Gutierrez-Rodrigues et al., 2014), the average telomere length in Z4sh-ESCs was comparable with wild-type ESCs (Figure S6C). This could be because cells with extremely short telomeres, which are a minor population that are supposed to be rejuvenated by the transient activation of *Zscan4*, are prone to die in Z4sh-ESCs without the benefit of telomere elongation (Figure 6E) and thus were excluded from the count, while the majority of the population, in which *Zscan4* is not activated anyway, was dominantly counted for the assay. Indeed when the lengths of the telomeres in individual cells were measured by flow-FISH, the population with normal telomere length was not so much affected by the knockdown of *Zscan4*. Instead, there was an increase in the population with extremely short telomeres in the knocked down cells (Figure 6A, 1.2% and 4.2% in Wt and Z4sh, respectively), which was reflected in the average telomere length. Taken together, we speculate that *Zscan4* is activated in response to telomere shortening, which may lead to genomic instability, and without sufficient expression of *Zscan4*, ESCs cannot divide.

In summary, we interpret correlation of *Zscan4* activity with cell-cycle length as follows: (1) the telomere becomes short after each cell division due to end replication problems; (2) then *Zscan4* becomes active sensing shortened telomeres; (3) activation of *Zscan4* elongates the telomeres, and meanwhile the cell cycle lengthens; (4) after the repair of the telomere, the cell cycle shortens and *Zscan4* becomes silent in the next cell cycle (Figure 7B).

DISCUSSION

In this study, we showed that *Zscan4* is activated independent of the pluripotent status represented by *Rex1* activity. On the other hand, increasing evidence from recent studies suggests involvement of the maintenance of telomere length in pluripotency (Hoffmeyer et al., 2012; Huang et al., 2011; Pucci et al., 2013; Wong et al., 2010). It is also shown that exogenous expression of *Zscan4* gives more stable pluripotency during embryogenesis (Amano et al., 2013) and also enhances the efficiency of iPSC generation (Hirata et al., 2012). If *Zscan4* is irrelevant to pluripotency, under what mechanisms can these be explained? As described by Zalzman et al. (2010), *Zscan4* contributes not only to telomere elongation but also to genomic stability. We interpret the contribution of *Zscan4* to more stable chimeric contribution (Amano et al., 2013) or higher efficiency of iPSC generation (Hirata et al., 2012) as the consequence of more stable proliferation of pluripotent ESCs owing to the activation of *Zscan4*, as shown by the short and stable cell cycles of Z4ex-ESCs in our experiments. Reversely, the low chimeric contribution by telomerase-deficient ESCs (Huang et al., 2011) may be a consequence of higher incidence of cell death in the short telomere ESCs as shown in our Z4sh-ESCs.

If the function of *Zscan4* is to elongate the telomeres, why is it co-expressed with telomerase? At the time of cell division, cells are exposed not only to telomere shortening

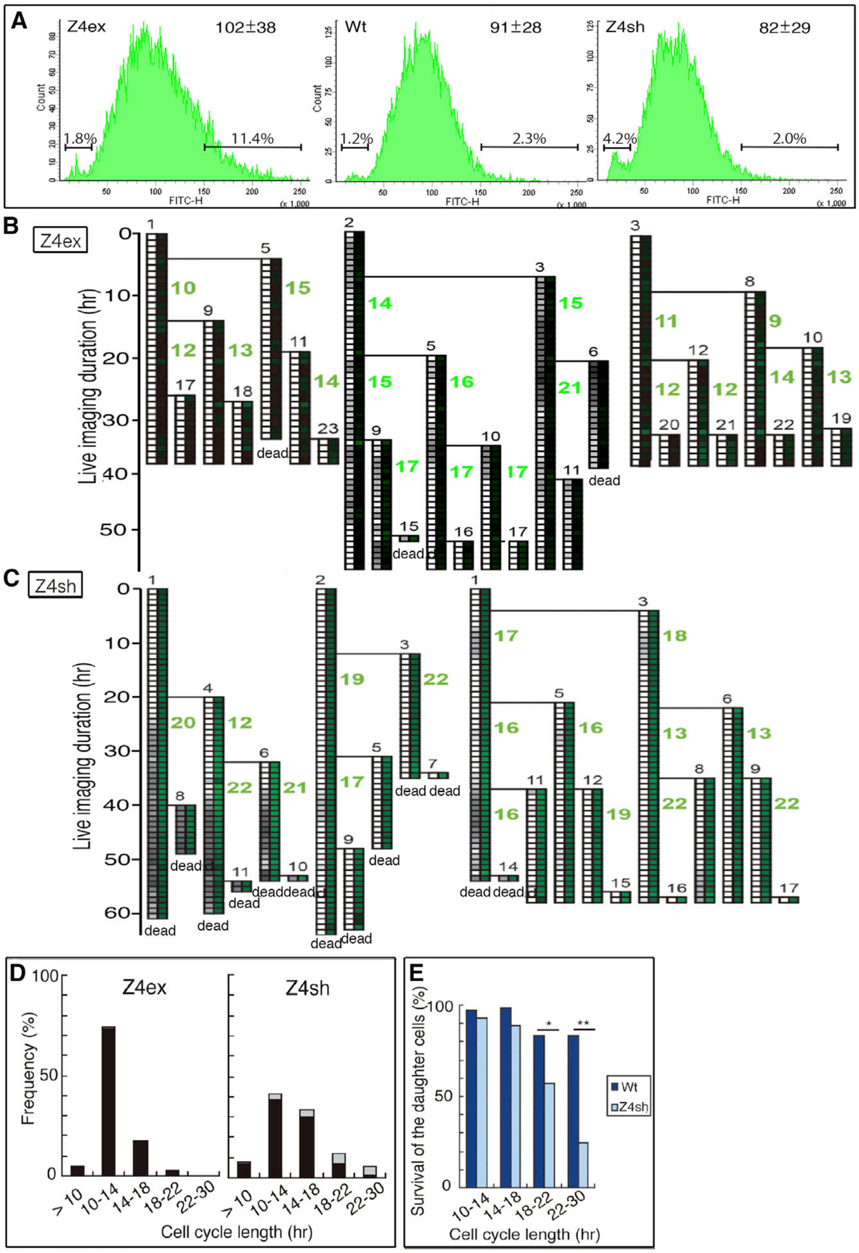

Figure 6. Expression Levels of *Zscan4* Affects the Cell-Cycle Length

(A) Telomere lengths of Z4ex-, wild-type or Z4sh-ESCs were measured by flow-FISH using an FITC-conjugated telomere probe. The average telomere intensity (×1,000) per cell ±SD is indicated. n > 7,000 cells. Two technical replicates showed the same tendency. Note that cells with longer telomeres were increased in Z4ex-ESCs compared with wild-type ESCs (11.4% vs 2.3%) and cells with shorter telomeres were increased in Z4sh-ESCs compared with wild-type ESCs (4.2% vs 1.2%). See also Figures S6B and S6C.

(B and C) Examples of the lineage trees of Z4ex-ESCs (B) and Z4sh-ESCs (C) with *Rex1*-Luciferase and *Zscan4*-Gal4-*UAS*-EGFP probes. The green scale on the right side of each lineage indicates the intensities of the *Zscan4*-Gal4-*UAS*-EGFP. Note that Z4ex-ESCs showed stable cell cycles without strong *Zscan4* activities (B), while Z4sh-ESCs showed a longer cell cycle with high basal EGFP expression (C).

(D) Histograms of the cell-cycle length of Z4ex- and Z4sh-ESCs (n = 194 and 289 cell divisions in three and 11 lineages from two independent experiments, respectively). Z4ex-ESCs increased in cells with shorter cell cycles. Black, the cells whose daughter cells divided within 30 hr; gray, the cells whose daughter cells died or did not divide within 30 hr.

(E) Equivalent of the histograms of the wild-type and Z4sh-ESCs from Figures 1B and (D), respectively, highlighting the difference in the survival rate of the daughter cells, i.e., black portion divided by black + gray. Student's t test was used for statistical analysis. *p < 0.01, **p < 0.005. See also Figure S7.

attributed to the end replication problem but also to DNA replication stress by which a DNA damage response is activated (Mazouzi et al., 2014). Also, telomere shortening beyond the threshold can activate a DNA damage response (Blackburn, 2001). It has been shown that telomere recovery and DNA repair share common mechanisms (Maser and DePinho, 2004; Doksani and de Lange, 2014). *Zscan4* may contribute to the repair of largely damaged telomeres caused by DNA replication stress, which may be the case homologous recombination is more efficient than telomerase-mediated telomere synthesis. This may also explain the characteristically transient and population restricted activation of *Zscan4*. Indeed, artificial DNA damage induced by reagents such as zeocin, cisplatin (Storm et al., 2014), and doxorubicin (Y.N.-F. and H.N., unpublished data) led to strong activation of *Zscan4*. If *Zscan4* is upregulated in response to DNA damage, this may also explain the expression of *Zscan4* at longer cell cycles as generally DNA damage response leads to cell-cycle elongation to repair the damaged DNA after corresponding check points (Harper and Elledge, 2007). In this study, we showed that longer cell cycles in ESCs were accompanied by a

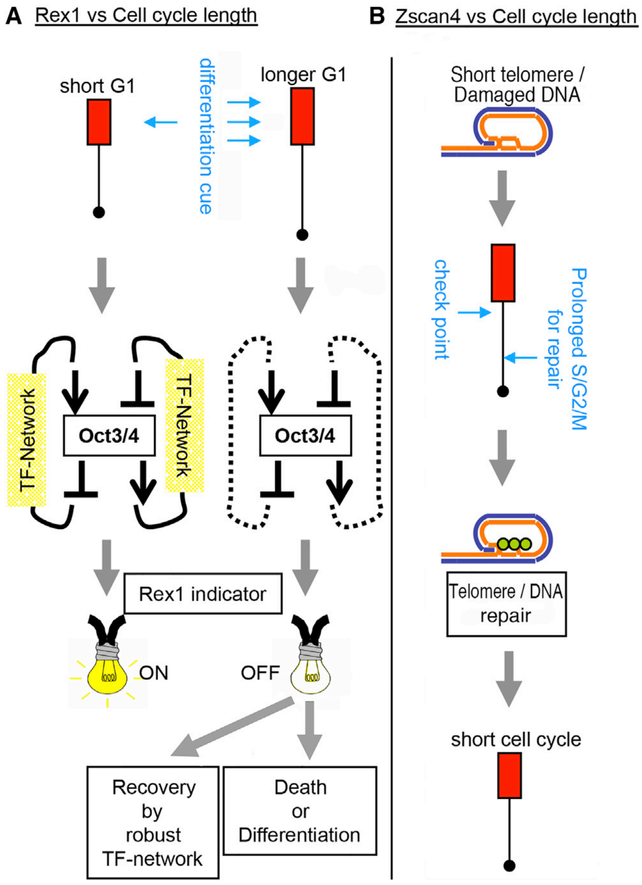

Figure 7. *Rex1* and *Zscan4* Correlate with the Cell-Cycle Length Under Different Mechanisms

(A) ESCs are continuously exposed to differentiation factors. For example, a very general growth factor, fibroblast growth factor (FGF), is always around ESCs as it is secreted by ESCs themselves (Kunath et al., 2007) and may also be contained in the media if supplemented with serum. Downstream of FGF, MAPK is activated, which suppresses pluripotent factors such as *Tbx3* and *Nanog* (Niwa et al., 2009). But ESCs form a robust transcription factor (TF) network that gradually adjusts the pluripotent status when the balance among the TFs becomes chaotic (Niwa et al., 2009). Since the battle between differentiation and pluripotency occurs at the transcription level, the conditions at the G1 phase should be the platform (Pauklin and Vallier, 2013). Thus, when the G1 phase (red box in this figure) is long, there are higher chances for the differentiation cues to invade, which then disturbs the TF network so the expression level of the master gene *Oct3/4* becomes altered and, as a consequence, the indicator *Rex1* turns off. Although *Rex1* is off, this should be distinguished from complete differentiation, because even at the longer cell cycles discussed here, the G1 phase remains in a typical ESC-like proportion (Figure 1D), and both the cell-cycle length (Figures 1A, 2C, and S5) and *Rex1* expression (Figure S3A) are reversible. In conventional culture (i.e., in serum-containing medium supplemented with LIF), ESCs are probably fluctuating between the two statuses illustrated on the left and right in (A). At longer cell cycles, the chances of invasion by differentiation cues can be higher, resulting in negative correlation of *Rex1* and the cell-cycle length (Figure S3B).

(B) In the event of telomere shortening beyond the threshold or DNA damage, DNA replication is paused until the repair is complete, which results in a prolonged S/G2/M phase. This probably underlies the activation of *Zscan4* at longer cell cycles (Figures 4A and 4B).

prolonged S/G2/M phase, and our previous study showed that activation of *Zscan4* induces arrest at the G2/M phase (Fujii et al., 2015), which is the phase for DNA repair. Also a recent study showed that activation of *Zscan4* induces heterochromatin decondensation, which permits DNA repair (Akiyama et al., 2015). If *Zscan4* is activated in response to DNA damage, this could also underlie the cell death observed in ESCs with hyperactive *Zscan4* that was shown in our live imaging, as these ESCs may have unbearable levels of DNA damage, by which they were eliminated.

Our previous study identified *Dax1* as a suppressor of *Zscan4* (Fujii et al., 2015). In *Dax1*-null ESCs, *Zscan4* was hyperactive and had a higher incidence of cell death, which was suppressed by the restoration of *Dax1*. This clearly indicates the significance of *Zscan4* suppression in ESC survival, which suggests that *Zscan4* can be a cause of cell death. Probably in *Dax1*-null ESCs, *Zscan4* was more sensitive to telomere shortening or DNA damage due to the lack of suppression by *Dax1*, and thus became hyperactive. Generally, too strong DNA damage response results in apoptosis (Harper and Elledge, 2007). Similarly, we speculate that hyperactive *Zscan4* activates signal cascades leading to cell death. In other words, at normal expression levels, *Zscan4* functions for telomere elongation and genomic stability, however when it is hyper-expressed, *Zscan4* can be a cause of cell death.

Zscan4 is sometimes considered as a marker of highly pluripotent status, merely due to its expression at the two-cell stage during development (Cerulo et al., 2014). However, based on our observation, we would like to propose that the expression of *Zscan4* is activated in response to telomere shortening and maybe to DNA damage, independent of the expression of the pluripotency-associated transcription factors. Indeed, a previous study has shown that *Zscan4* can respond to artificial telomere shortening without affecting the expression of *Oct3/4* (Huang et al., 2011). Thus it should be more reasonable to consider that *Zscan4* is activated merely for the physical maintenance of the genome and does not necessarily represent the two-cell-stage-like status of ESCs, the status in which ESCs have the potential to develop into both embryonic and extra-embryonic lineages (Macfarlan et al., 2012). Then if the function of *Zscan4* is restricted to telomere elongation and genomic stability, why is *Zscan4* specifically expressed at the two-cell stage in vivo? One bold idea could be that two-cell-stage embryos have a special

mechanism to recover from telomere shortening after meiosis, otherwise the telomeres will be short generation after generation. And maybe for this special mission required for just one cell division, *Zscan4* is expressed. In the inner cell mass of the blastocyst, from where ESCs derive, this mechanism might be silent or just invisible, and may become visible in long-term culture.

By retrospective analysis of the lineage tree, we were able to elucidate the expression pattern of *Zscan4* in terms of the correlation with the cell-cycle length. *Zscan4* was activated regardless of the pluripotent status probably sensing shortened telomeres and genomic instability. Maybe the physiological regulation of pluripotency and physical maintenance of the genome should be considered separately. If live imaging of telomere shortening and/or manipulation of the telomere length was possible, this should give more direct evidence, but for now it is technically difficult and will be our next challenge. This study visualized the fluctuation of the cell-cycle length and showed one of the mechanisms to maintain self-renewability in ESCs opposing naturally occurring stem cell aging.

EXPERIMENTAL PROCEDURES

Cell Culture

EB5 ESCs (derived from male E14tg2a ESCs) were cultured on a gelatin-coated culture dish in Glasgow minimum essential medium supplemented with 10% FCS, 1× sodium pyruvate, 1× non-essential amino acids, 0.1 mM 2-mercaptoethanol, and 1,000 U/ml of LIF. For 2i/LIF culture, NDiff227 medium (Stem Cells) was supplemented with 1,000 U/ml LIF, 3 μM GSK3β-inhibitor CHIR99021 (Stemgent), and 1 μM MEK inhibitor PD0325901 (Stemgent).

Generation of *Rex1p*-Luc/*Zscan4p*-Gal4-*UAS*-EGFP ESC

Luciferase2 was inserted downstream of the *Rex1* promoter in one of the alleles by a Cre-loxP mediated cassette exchange system based on the system we previously reported modified for manipulation of the *Rex1* allele (Masui et al., 2005). Briefly, host cells were generated by electroporation of EB5 ESCs using Gene Pulser (Bio-Rad) with a linearized vector possessing a *loxP-IRESneo-pA:PGKpacΔtkpA-loxPV* sequence flanked by homologous arms targeting the *Rex1* ORF. Then a plasmid vector possessing a translational amplifier from the 5′ UTR of the homeobox gene *Gtx* (super IRES) that was cut out from pGTIV3 vector (a kind gift from Dr. Joshua Brickman) (Tsakiridis et al., 2009) fused to the 5′ of *Luciferase2* that was cut out from pGL4.10 vector (Promega) flanked by *loxP* and a mutant *loxPV* (Figure S2A) was co-transfected with a pCAGGS-Cre vector using Lipofectoamine 2000 (Invitrogen), followed by selection with 1 μM gancyclovir to obtain *Rex1p*-Luc ESCs.

Then the *Rex1p*-Luc ESCs were transfected with a *piggyBac* transposon vector coding the *Zscan4c* promoter (amplified from the EB5 genomic DNA) and the *trans*-activating domain of *Gal4* (Figure 2A), together with the *piggyBac* vectors pPB-UAS-hCMV promoter-EGFP and pPB-H2BmCherry-IRESpac, followed by selection with puromycin to obtain *Rex1p*-Luc ESCs stably transfected with *Zscan4p-Gal4TD*, *UAS-EGFP*, and *H2B-mCherry*.

Generation of Z4ex-ESC and Z4sh-ESC

For generation of Z4ex-ESCs, EB5 or *Rex1p*-Luc ESCs were transfected with a *piggyBac* vector coding *Zscan4c* (amplified from cDNA pool derived from wild-type mouse ESCs) downstream of the Tet-responsible element with a minimal *CMV* promoter (*hCMV*1*) (Figure S6A), together with the *piggyBac* vectors pPB-CAG-rtTA-IRESneo and pPB-H2BmCherry-IRESpac, followed by selection with G418 and puromycin to obtain ESCs stably transfected with Tet-inducible *Zscan4c*, *rtTA*, and *H2B-mCherry*.

For generation of Z4sh-ESCs, shRNA was designed based on the 19-nucleotide shRNA sequence previously identified (Zalzman et al., 2010): 5′-<ATT GTG AGA CC>AAA AAA [CAG AAG CCT GGC ATT CCC T]AAG CTT [*AGG GAA TGC CAG GCT TCT G*]<GGT CTC ACA GG>−3′, that is 5′-<linker sense>AAA AAA[19-nucleotide sense] a hairpin loop [19-nucleotide antisense]<linker antisense>. After annealing with complementary sequence, the shRNA was cloned into the *miR155* region of a *piggyBac* vector that was modified from BLOCK-iT miR155 expression vector (Invitrogen) (Adachi et al., 2013), which now has a Tet-responsible element with a minimal *CMV* promoter (Figure S6A). The resulting vector was transfected into EB5 or *Rex1p*-Luc ESCs together with the *piggyBac* vectors pPB-CAG-rtTA-IRESneo and pPB-H2BmCherry-IRESpac, followed by selection with G418 and puromycin to obtain ESCs stably transfected with Tet-inducible *Zscan4-shRNA*, *rtTA*, and *H2B-mCherry*.

Live Imaging

ESCs were seeded 1,000 cells per well on a thin plastic-bottom eight-well chamber (Ibidi) coated with Laminin511 (1 μg/cm^2; Nippi) in FCS/LIF medium. 2i/LIF medium was also used for some experiments. Cells were monitored on a stage incubator in a humid atmosphere and 5% CO_2 at 37°C (Tokai Hit) under an inverted microscope (IX81; Olympus) equipped with MetaMorph imaging software (Molecular Devices).

For simultaneous monitoring of chemiluminescence and fluorescence, the IX81 microscope was modified to shut out all the lights from the mechanics of the microscope (Olympus), tightly covered with a light-shielding tent (Hamamatsu Photonics), and equipped with a CCD camera (ImagEM; Hamamatsu Photonics) and an LED illuminator (Olympus). Chemiluminescence was induced by addition of 1 mM Luciferin (Wako) to the medium and was detected by opening the shutter for 5 min with an electron-multiplying (EM) gain of 255 and the CCD resolution set at binning = 1 or 2. Fluorescence was detected by exposure for 300–500 ms with EM gain of 100. Images were taken every 1 hr with a 20× objective lens.

Fucci-Based Cell-Cycle Analysis

Fucci vectors (provided by Dr. Atushi Miyawaki) (Sakaue-Sawano et al., 2008) were modified by fusing the two elements, *Fucci-G1-Kusabira_orange* and *Fucci-S/G2/M-Azami_green* with P2A. The resulting *Fucci-G1-KO-P2A-S/G2/M-AG* sequence was inserted into a *piggyBac* vector cassette under the control of *CAG*

promoter and was transfected together with pPB-H2BCFP-IRESpac, followed by selection with puromycin to obtain stably transfected ESCs. Cells were monitored under the IX81 microscope equipped with a stage incubator, a confocal spinning disk (CSU-X1; Yokogawa), a CCD camera (iXon; Andor), and a laser illuminator (LMM5; Spectral) with wavelength 448, 488, and 561 nm, an exposure time of 500 ms, and EM gain of 300. Time-lapse images were taken every 15 min or 30 min with a 20× objective lens.

Lineage Tracking

Each cell was tracked manually by drawing a region surrounding the nuclear marker H2B-mCherry (Figure 2B). The average intensities of the chemiluminescence and fluorescence were measured using MetaMorph imaging software. The intensity data were manually converted to a Microsoft Excel sheet in the format of the lineage. Then the data were used as input for a VBA macro (input data and source code are provided in Data S1) that was programmed to automatically draw a lineage tree with the intensity color scale (Figures S2E and 3A) in Microsoft PowerPoint.

qPCR for mRNA Expression Level

First-strand DNA was synthesized from total RNA prepared by a QuickGene RNA cultured cell HC kit (KURABO) in 20 μl of the reaction mixture containing oligo-dT primers using a ReverTra Ace first-strand synthesis kit (Toyobo). Real-time PCR was performed with THUNDERBIRD SYBR qPCR Mix (Toyobo) using a CFX384 Real-Time System (Bio-Rad). The expression level of *Gapdh* was used as an internal control. The average of triplicate reactions was calculated. Sequences of primer pairs are as follows:

Zscan4c$_{endo+exo}$ (Zalzman et al., 2010)
forward 5′-GAGATTCATGGAGAGTCTGACTGATGAGTG-3′
reverse 5′-GCTGTTGTTTCAAAAGCTTGATGACTTC-3′
Zscan4c$_{sh\text{-}target}$
forward 5′-ATTCTCTACAGTGTTCTTGAC-3′
reverse 5′-CTAAGACTTGGGATGAAAAC-3′
Gapdh
forward 5′-ACCACAGTCCATGCCATCAC-3′
reverse 5′-TCCACCACCCTGTTGCTGTA-3′

CFSE Dilution Assay

Cells were labeled with 20 μM CFSE dye (Wako) for 15 min at 37°C, followed by centrifugation and two washes in PBS, and cultured for 48 hr. After 48 hr, the cells were trypsinized, centrifuged, and washed twice, then suspended in Hanks buffer with 1% BSA and subjected to FACS sorting by AriaIII (BD Biosciences). Cells labeled just before sorting and unlabeled cells were used as positive and negative controls, respectively.

Telomere Length Measurement by Flow-FISH

A telomere PNA kit/fluorescein isothiocyanates (FITC) for flow cytometry (Dako, K5327) was used following the manufacturer's protocol. Briefly, 10^6 cells were hybridized with an FITC-conjugated telomere PNA probe at 82°C for 5 min and incubated at room temperature overnight, followed by DNA staining with propidium iodide. For cells that were collected after the CFSE dilution assay, 5×10^5 cells were hybridized with 150 ng/ml Cy3-conjugated telomere PNA probe (TelG-Cy3, Panagene) instead of the FITC telomere PNA probe provided in the kit, because carry over of CFSE dye with wavelength close to that of FITC was still detected after in situ hybridization. The intensities of telomere-bound fluorescence (either FITC or Cy3) were analyzed by FACS with AriaIII (BD Biosciences).

SUPPLEMENTAL INFORMATION

Supplemental Information includes Supplemental Experimental Procedures, seven figures, and one data file and can be found with this article online at http://dx.doi.org/10.1016/j.stemcr.2016.02.010.

AUTHOR CONTRIBUTIONS

Y.N.-F. designed and performed the experiments, analyzed the data, and wrote the manuscript. H.N. generated the plasmids and gave scientific guidance.

ACKNOWLEDGMENTS

We thank Dr. Joshua Brickman (University of Copenhagen) for providing us with the plasmid pGTIV3, and Dr. Atsushi Miyawaki (RIKEN BSI) for providing us with the Fucci vector. We also thank Dr. Mariko Yamane for technical support with FACS sorting, and Dr. Akira Futatsugi for critical reading. This work was supported by a RIKEN research grant and the CREST program from the Japan Science and Technology Agency (JST) to H.N.

Received: September 18, 2015
Revised: February 12, 2016
Accepted: February 12, 2016
Published: March 17, 2016

REFERENCES

Adachi, K., Nikaido, I., Ohta, H., Ohtsuka, S., Ura, H., Kadota, M., Wakayama, T., Ueda, H.R., and Niwa, H. (2013). Context-dependent wiring of Sox2 regulatory networks for self-renewal of embryonic and trophoblast stem cells. Mol. Cell *52*, 380–392.

Akiyama, T., Xin, L., Oda, M., Sharov, A.A., Amano, M., Piao, Y., Cadet, J.S., Dudekula, D.B., Qian, Y., Wang, W., et al. (2015). Transient bursts of Zscan4 expression are accompanied by the rapid derepression of heterochromatin in mouse embryonic stem cells. DNA Res. *22*, 307–318.

Amano, T., Hirata, T., Falco, G., Monti, M., Sharova, L.V., Amano, M., Sheer, S., Hoang, H.G., Piao, Y., Stagg, C.A., et al. (2013). Zscan4 restores the developmental potency of embryonic stem cells. Nat. Commun. *4*, 1966.

Baerlocher, G.M., Vulto, I., de Jong, G., and Lansdorp, P.M. (2006). Flow cytometry and FISH to measure the average length of telomeres (flow FISH). Nat. Protoc. *1*, 2365–2376.

Blackburn, E.H. (2001). Switching and signaling at the telomere. Cell *106*, 661–673.

Callicott, R.J., and Womack, J.E. (2006). Real-time PCR assay for measurement of mouse telomeres. Comp. Med. *56*, 17–22.

Carter, M.G., Stagg, C.A., Falco, G., Yoshikawa, T., Bassey, U.C., Aiba, K., Sharova, L.V., Shaik, N., and Ko, M.S. (2008). An in situ hybridization-based screen for heterogeneously expressed genes in mouse ES cells. Gene Expr. Patterns *8*, 181–198.

Cerulo, L., Tagliaferri, D., Marotta, P., Zoppoli, P., Russo, F., Mazio, C., DeFelice, M., Ceccarelli, M., and Falco, G. (2014). Identification of a novel gene signature of ES cells self-renewal fluctuation through system-wide analysis. PLoS One *9*, e83235.

Chambers, I., Silva, J., Colby, D., Nichols, J., Nijmeijer, B., Robertson, M., Vrana, J., Jones, K., Grotewold, L., and Smith, A. (2007). Nanog safeguards pluripotency and mediates germline development. Nature *450*, 1230–1234.

Doksani, Y., and de Lange, T. (2014). The role of double-strand break repair pathways at functional and dysfunctional telomeres. Cold Spring Harb Perspect. Biol. *6*, a016576.

Falco, G., Lee, S.L., Stanghellini, I., Bassey, U.C., Hamatani, T., and Ko, M.S. (2007). Zscan4: a novel gene expressed exclusively in late 2-cell embryos and embryonic stem cells. Dev. Biol. *307*, 539–550.

Fujii, S., Nishikawa-Torikai, S., Futatsugi, Y., Toyooka, Y., Yamane, M., Ohtsuka, S., and Niwa, H. (2015). Nr0b1 is a negative regulator of Zscan4c in mouse embryonic stem cells. Sci. Rep. *5*, 9146.

Gutierrez-Rodrigues, F., Santana-Lemos, B.A., Scheucher, P.S., Alves-Paiva, R.M., and Calado, R.T. (2014). Direct comparison of flow-FISH and qPCR as diagnostic tests for telomere length measurement in humans. PLoS One *9*, e113747.

Harper, J.W., and Elledge, S.J. (2007). The DNA damage response: ten years after. Mol. Cell *28*, 739–745.

Hayashi, K., Lopes, S.M., Tang, F., and Surani, M.A. (2008). Dynamic equilibrium and heterogeneity of mouse pluripotent stem cells with distinct functional epigenetic status. Cell Stem Cell *3*, 391–401.

Hirata, T., Amano, T., Nakatake, Y., Amano, M., Piao, Y., Hoang, H.G., and Ko, M.S. (2012). Zscan4 transiently reactivates early embryonic genes during the generation of induced pluripotent stem cells. Sci. Rep. *2*, 208.

Hoffmeyer, K., Raggioli, A., Rudloff, S., Anton, R., Hierholzer, A., Del Valle, I., Hein, K., Vogt, R., and Kemler, R. (2012). Wnt/β-catenin signaling regulates telomerase in stem cells and cancer cells. Science *336*, 1549–1554.

Huang, J., Wang, F., Okuka, M., Liu, N., Ji, G., Ye, X., Zuo, B., Li, M., Liang, P., Ge, W.W., et al. (2011). Association of telomere length with authentic pluripotency of ES/iPS cells. Cell Res. *21*, 779–792.

Iyer, M., Wu, L., Carey, M., Wang, Y., Smallwood, A., and Gambhir, S.S. (2001). Two-step transcriptional amplification as a method for imaging reporter gene expression using weak promoters. Proc. Natl. Acad. Sci. USA *98*, 14595–14600.

Kunath, T., Saba-El-Leil, M.K., Almousailleakh, M., Wray, J., Meloche, S., and Smith, A. (2007). FGF stimulation of the Erk1/2 signalling cascade triggers transition of pluripotent embryonic stem cells from self-renewal to lineage commitment. Development *134*, 2895–2902.

Lansdorp, P.M., Verwoerd, N.P., van de Rijke, F.M., Dragowska, V., Little, M.T., Dirks, R.W., Raap, A.K., and Tanke, H.J. (1996). Heterogeneity in telomere length of human chromosomes. Hum. Mol. Genet. *5*, 685–691.

Macfarlan, T.S., Gifford, W.D., Driscoll, S., Lettieri, K., Rowe, H.M., Bonanomi, D., Firth, A., Singer, O., Trono, D., and Pfaff, S.L. (2012). Embryonic stem cell potency fluctuates with endogenous retrovirus activity. Nature *487*, 57–63.

Maser, R.S., and DePinho, R.A. (2004). Telomeres and the DNA damage response: why the fox is guarding the henhouse. DNA Repair (Amst) *3*, 979–988.

Masui, S., Shimosato, D., Toyooka, Y., Yagi, R., Takahashi, K., and Niwa, H. (2005). An efficient system to establish multiple embryonic stem cell lines carrying an inducible expression unit. Nucleic Acids Res. *33*, e43.

Masui, S., Ohtsuka, S., Yagi, R., Takahashi, K., Ko, M.S., and Niwa, H. (2008). Rex1/Zfp42 is dispensable for pluripotency in mouse ES cells. BMC Dev. Biol. *8*, 45.

Mazouzi, A., Velimezi, G., and Loizou, J.I. (2014). DNA replication stress: causes, resolution and disease. Exp. Cell Res. *329*, 85–93.

Nakai-Futatsugi, Y., and Niwa, H. (2013). Transcription factor network in embryonic stem cells: heterogeneity under the stringency. Biol. Pharm. Bull. *36*, 166–170.

Nichols, J., Zevnik, B., Anastassiadis, K., Niwa, H., Klewe-Nebenius, D., Chambers, I., Schöler, H., and Smith, A. (1998). Formation of pluripotent stem cells in the mammalian embryo depends on the POU transcription factor Oct4. Cell *95*, 379–391.

Niwa, H., Miyazaki, J., and Smith, A.G. (2000). Quantitative expression of Oct-3/4 defines differentiation, dedifferentiation or self-renewal of ES cells. Nat. Genet. *24*, 372–376.

Niwa, H., Ogawa, K., Shimosato, D., and Adachi, K. (2009). A parallel circuit of LIF signaling pathways maintains pluripotency of mouse ES cells. Nature *460*, 118–122.

Pauklin, S., and Vallier, L. (2013). The cell-cycle state of stem cells determines cell fate propensity. Cell *155*, 135–147.

Pauklin, S., Pedersen, R.A., and Vallier, L. (2011). Mouse pluripotent stem cells at a glance. J. Cell Sci. *124*, 3727–3732.

Pucci, F., Gardano, L., and Harrington, L. (2013). Short telomeres in ESCs lead to unstable differentiation. Cell Stem Cell *12*, 479–486.

Rufer, N., Dragowska, W., Thornbury, G., Roosnek, E., and Lansdorp, P.M. (1998). Telomere length dynamics in human lymphocyte subpopulations measured by flow cytometry. Nat. Biotechnol. *16*, 743–747.

Sakaue-Sawano, A., Kurokawa, H., Morimura, T., Hanyu, A., Hama, H., Osawa, H., Kashiwagi, S., Fukami, K., Miyata, T., Miyoshi, H., et al. (2008). Visualizing spatiotemporal dynamics of multicellular cell-cycle progression. Cell *132*, 487–498.

Singh, A.M., Hamazaki, T., Hankowski, K.E., and Terada, N. (2007). A heterogeneous expression pattern for Nanog in embryonic stem cells. Stem Cells *25*, 2534–2542.

Storm, M.P., Kumpfmueller, B., Bone, H.K., Buchholz, M., Sanchez Ripoll, Y., Chaudhuri, J.B., Niwa, H., Tosh, D., and Welham, M.J. (2014). Zscan4 is regulated by PI3-kinase and DNA-damaging agents and directly interacts with the transcriptional repressors LSD1 and CtBP2 in mouse embryonic stem cells. PLoS One *9*, e89821.

Toyooka, Y., Shimosato, D., Murakami, K., Takahashi, K., and Niwa, H. (2008). Identification and characterization of subpopulations in undifferentiated ES cell culture. Development *135*, 909–918.

Tsakiridis, A., Tzouanacou, E., Rahman, A., Colby, D., Axton, R., Chambers, I., Wilson, V., Forrester, L., and Brickman, J.M. (2009). Expression-independent gene trap vectors for random and targeted mutagenesis in embryonic stem cells. Nucleic Acids Res. *37*, e129.

White, J., and Dalton, S. (2005). Cell cycle control of embryonic stem cells. Stem Cell Rev. *1*, 131–138.

Wong, C.W., Hou, P.S., Tseng, S.F., Chien, C.L., Wu, K.J., Chen, H.F., Ho, H.N., Kyo, S., and Teng, S.C. (2010). Krüppel-like transcription factor 4 contributes to maintenance of telomerase activity in stem cells. Stem Cells *28*, 1510–1517.

Ying, Q.-L., Wray, J., Nichols, J., Batlle-Morera, L., Doble, B., Woodgett, J., Cohen, P., and Smith, A. (2008). The ground state of embryonic stem cell self-renewal. Nature *453*, 519–523.

Zalzman, M., Falco, G., Sharova, L.V., Nishiyama, A., Thomas, M., Lee, S.L., Stagg, C.A., Hoang, H.G., Yang, H.T., Indig, F.E., et al. (2010). Zscan4 regulates telomere elongation and genomic stability in ES cells. Nature *464*, 858–863.

Stem Cell Reports, Volume *6*

Supplemental Information

Zscan4 Is Activated after Telomere Shortening in Mouse Embryonic Stem Cells

Yoko Nakai-Futatsugi and Hitoshi Niwa

Supplemental Figures:

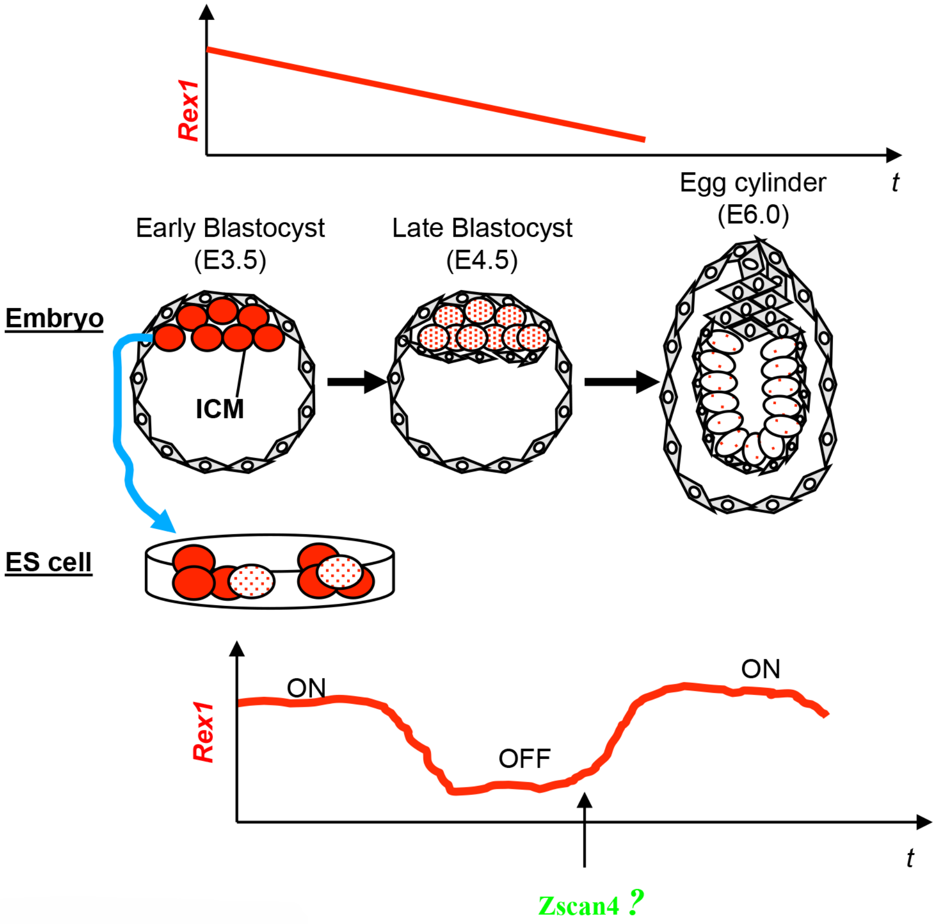

Figure S1, Related to Figure 2   Conceptual figure of the hypothesis

In vivo, the pluripotency-indicator *Rex1* is expressed only for 24 hours in the ICM during development, while *in vitro*, it is repeatedly up-regulated in mouse ESCs that derived from the ICM. To find the mechanism for this unnatural up-regulation, we will seek the possibility whether the "rejuvenation-factor" *Zscan4* plays the role.

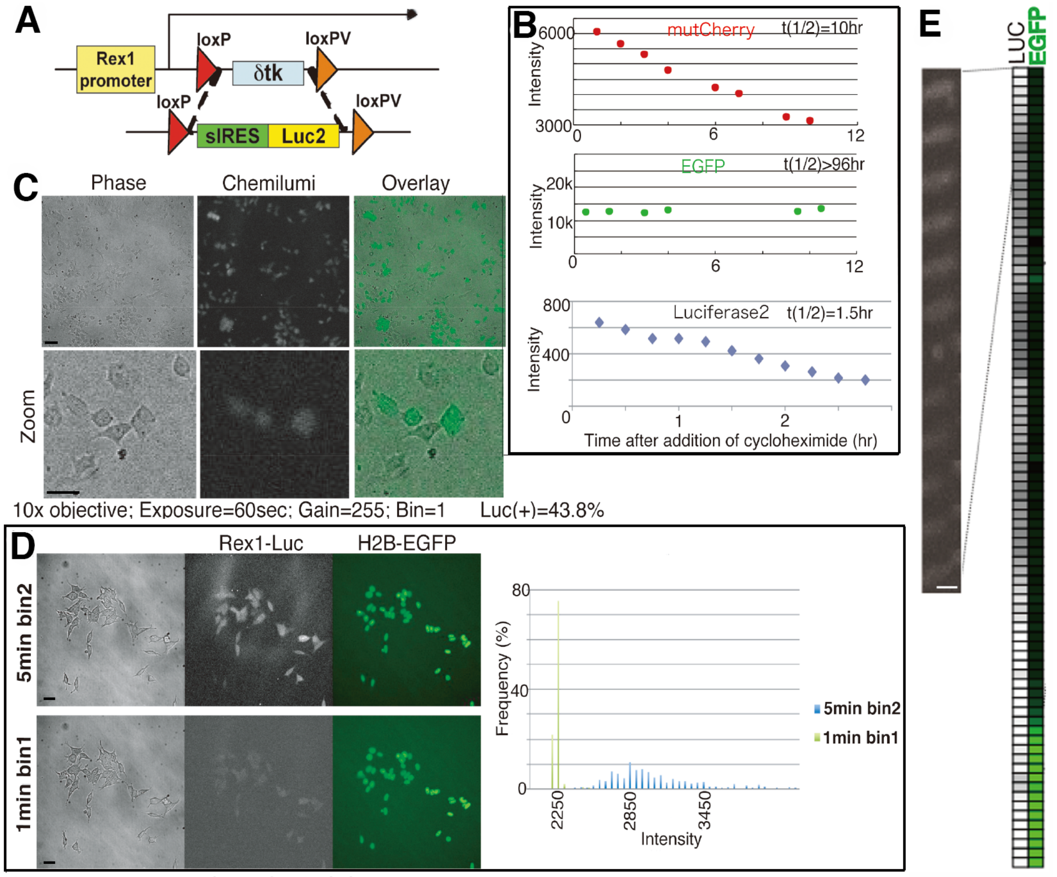

Figure S2, Related to Figure 2 Monitoring the *Rex1*-promoter activity

A Design of the exchange vector for *Rex1p*-Luc2. *Luciferase2* with "super-*IRES*" (Tsakiridis et al., 2009) fused on the 5' was flanked by *loxP* and a mutant *loxPV*, which was inserted by the cassette exchange system (Masui et al., 2005) into the downstream of the *Rex1* promoter of one of the alleles. **B** Half-lives of Luciferase2, EGFP, and muted mCherry were measured by the intensities of the images taken under the microscope following cycloheximide treatment. The half-life of Luciferase2 was as short as 1.5 hours while EGFP was very stable and even the fluorescent with the shortest half-life we were able to make, by adding mutated PEST domain (Li et al., 1998) to the N-terminal of monomeric Cherry, still had a much longer half-life. **C** Luciferase2, with its short half-life, allows monitoring of the kinetics of *Rex1*. *Rex1*-Luc showed 43% of Rex1-positive cells in culture, which was less than half compared to our previous study using *Rex1*-EGFP that showed 90% of *Rex1*-positive population (Toyooka et al., 2008). **D** Detection level of chemiluminescence depends on the exposure time. Exposure of 5 minutes gave clearer image (*left panel*) and broader distribution of intensity (*right panel*). This condition was used for all the experiments. **E** The intensity of the chemiluminescence (represents *Rex1* activity) and the intensity of GFP was converted to gray-scale and green-scale, respectively, using a handmade program written in Microsoft VBA. The actual image of the cell with slight down-regulation of *Rex1* is shown together with the gray-scale. Bars = 50μm (***C*** *upper*), 20μm (***C*** *lower* and ***D***) and 7.5μm (***E***).

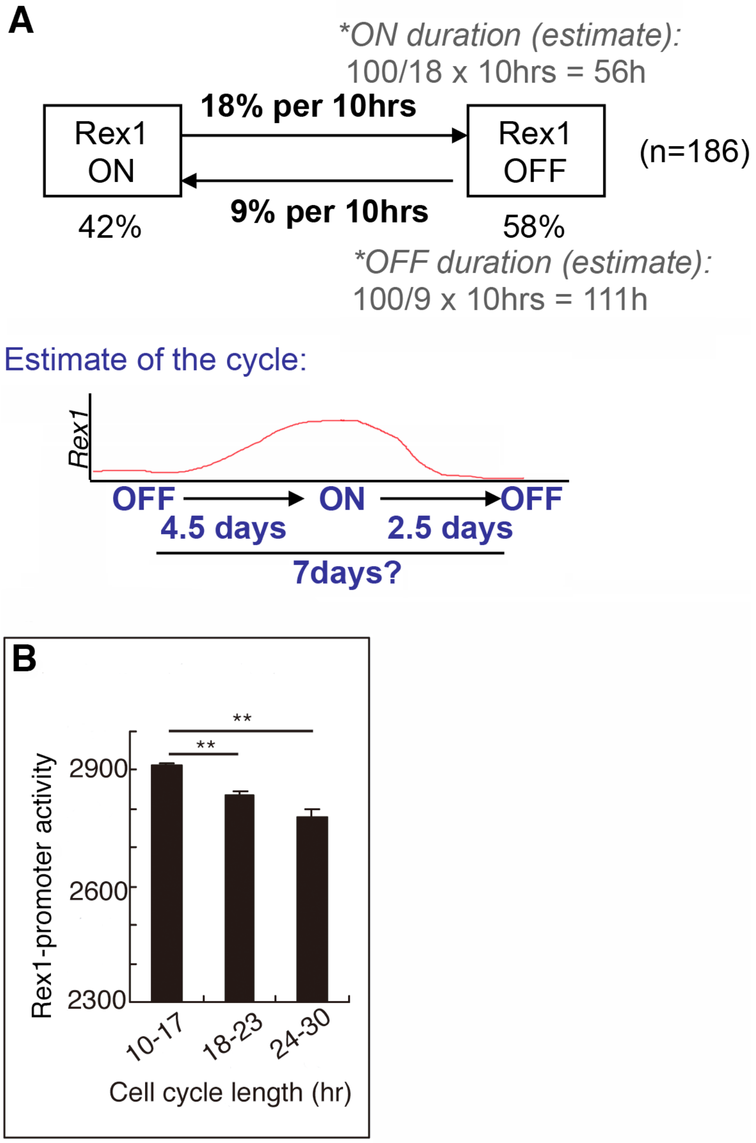

Figure S3, Related to Figure 2 ***Rex1* showed different kinetics from *Zscan4***

A Estimating the cycle length of the *Rex1*-fluctuation. By using the chemiluminescence Luciferase 2, the reversible up- and down-regulation of *Rex1* as previously predicted (Toyooka et al., 2008) was clearly visualized in the lineage tree (Figure 2C). However the cycle of the *Rex1*-fluctuation seemed very long, and it was not practical to measure the fluctuation by tracing the up and down repeat of one cell during the observation. So instead, many ESCs expressing *Rex1*-Luciferase were monitored for 10 hours under our live imaging system, and the number of the cells that turned from on to off and vice versa within 10 hours was used for the estimate. This rough estimate tells turning off seems to be easier than turning on, which is consistent with previous work showing that in the fluctuating wave of pluripotency, down-regulation of pluripotency-associated genes is faster than the up-regulation (Hayashi et al., 2008). In this estimate, we see the cycle of *Rex1*-fluctuation is not in the range of minutes or hours but of days or maybe of weeks. **B** *Rex1* promoter activity represented by *Rex1*-Luciferase intensity in the lineage tree was analyzed against the cell cycle length (n = 391 cells from 8 lineages from 2 experiments). At longer cell cycle, *Rex1* showed lower promoter activity. Bar graphs represent average with error bars of standard error. For statistical analysis, Student's T-test was used. **: $p < 0.005$.

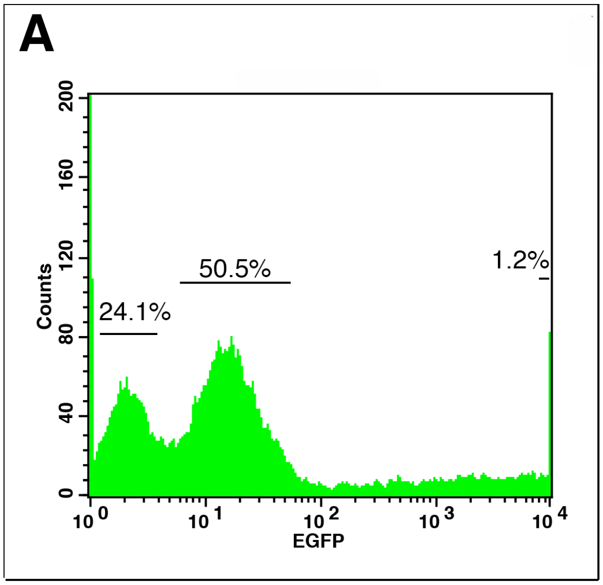

hours	GFPbk	GFP	log(GFP/bk)
50	7858.15	8114.24	0.013928
51	7885.28	8096.95	0.011504
52	7877.8	8161.81	0.015382
53	7884.08	8183.03	0.016163
54	7910.2	8165.38	0.013789
55	7923.36	8183.73	0.014042
56	7945.79	8214.46	0.014442

hours	GFPbk	GFP	log(GFP/bk)
69	7978.79	8696.33	0.037399
70	8010.56	9047.48	0.052865
71	7985.03	9710.52	0.084966
72	8012.77	10315.7	0.109716
73	8040.92	10706.3	0.124334
74	8037.16	11333.6	0.149265
75	8054.04	11935.9	0.170841

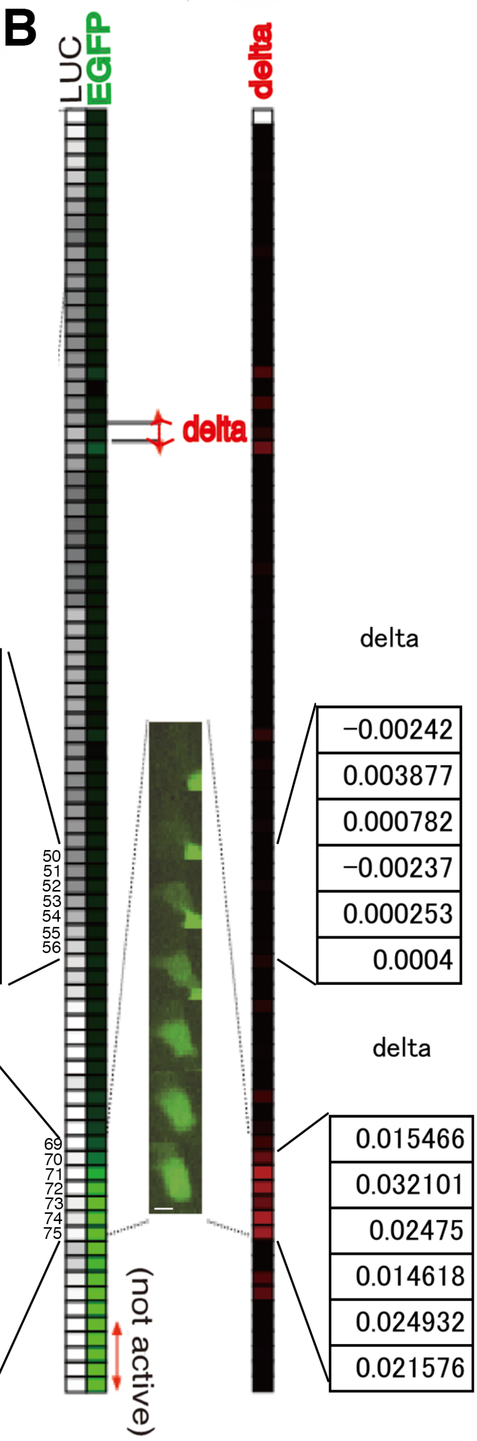

delta
−0.00242
0.003877
0.000782
−0.00237
0.000253
0.0004

delta
0.015466
0.032101
0.02475
0.014618
0.024932
0.021576

Figure S4, Related to Figures 2 and 3 **Detection of the *Zscan4*-pormoter activity by the Gal4-*UAS*-EGFP system**

A FACS analysis of the ESCs transfected with *Zscan4p*-Gal4 and *UAS*-EGFP vectors. There was 1.2% of the population with hyper-active *Zscan4* and notably 50.5% of weak positive population that was not detected in previous studies. **B** As the transactivator system amplified the GFP-intensity to a wide range, logarithm of the GFP-intensity subtracted by the logarithm of the background intensity was used to calculate the *delta* signal. The intention of using logarithm of the intensity was to fit the wide range of GFP-intensity into the 256-intensity-scale of the lineage tree, and also to adjust the effect of the fluctuation of the detection system (*i.e.* noise) that was higher at higher intensities. While positive *delta* indicates the promoter activity of *Zscan4*, considering the long half-life of EGFP, negative *delta* should indicate the degradation of the EGFP-protein and/or fluctuation of the detection system. Thus only the positive *delta* was used for the analysis in Figure 4. Bar = 7.5μm.

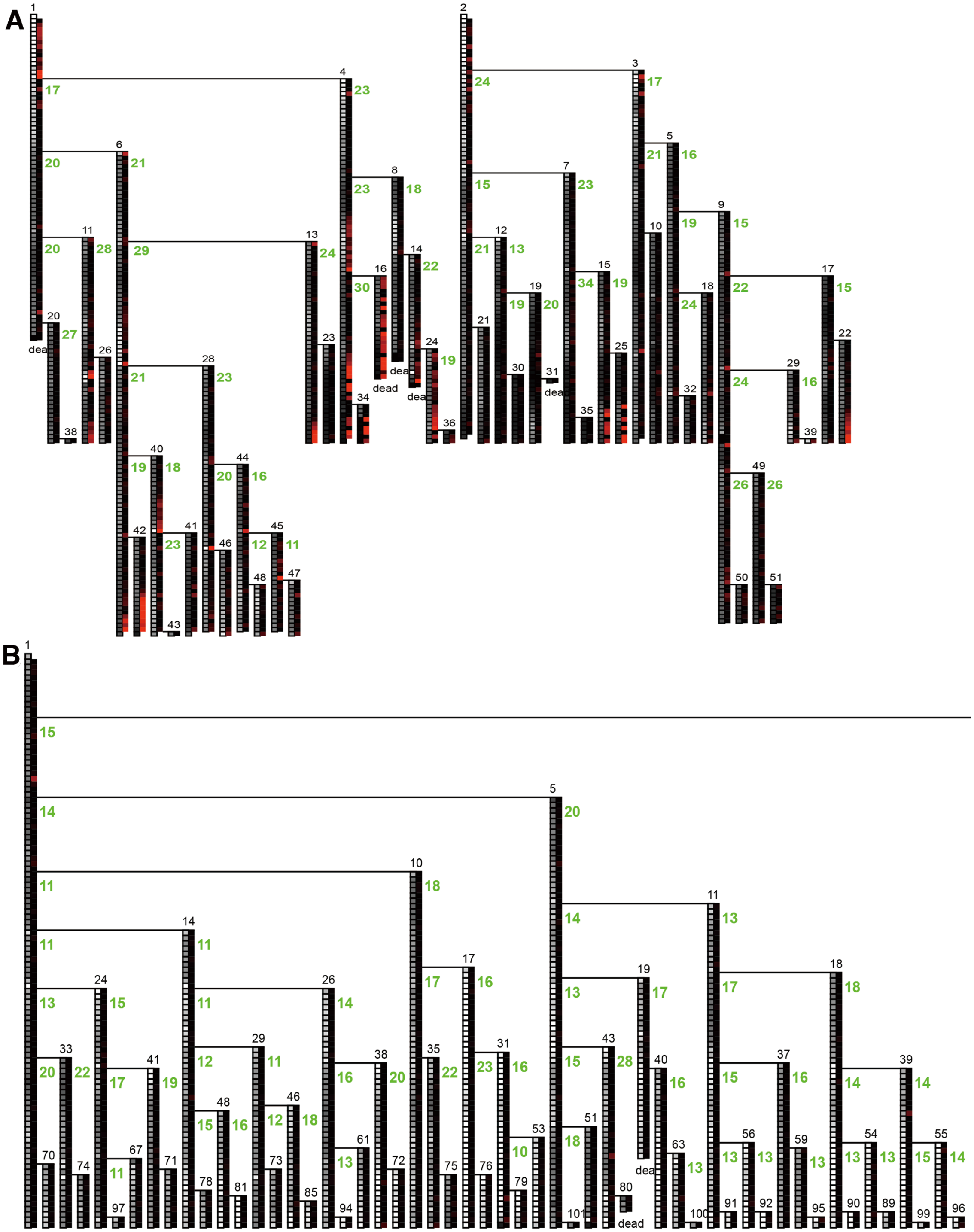

Figure S5, Related to Figure 3 **Two more examples of the lineage trees with *Rex1*-Luciferase and *delta* of *Zscan4*-Gal4-*UAS*-EGFP**

As shown in ***B***, *Zscan4* was less active when the cell cycle was stable.

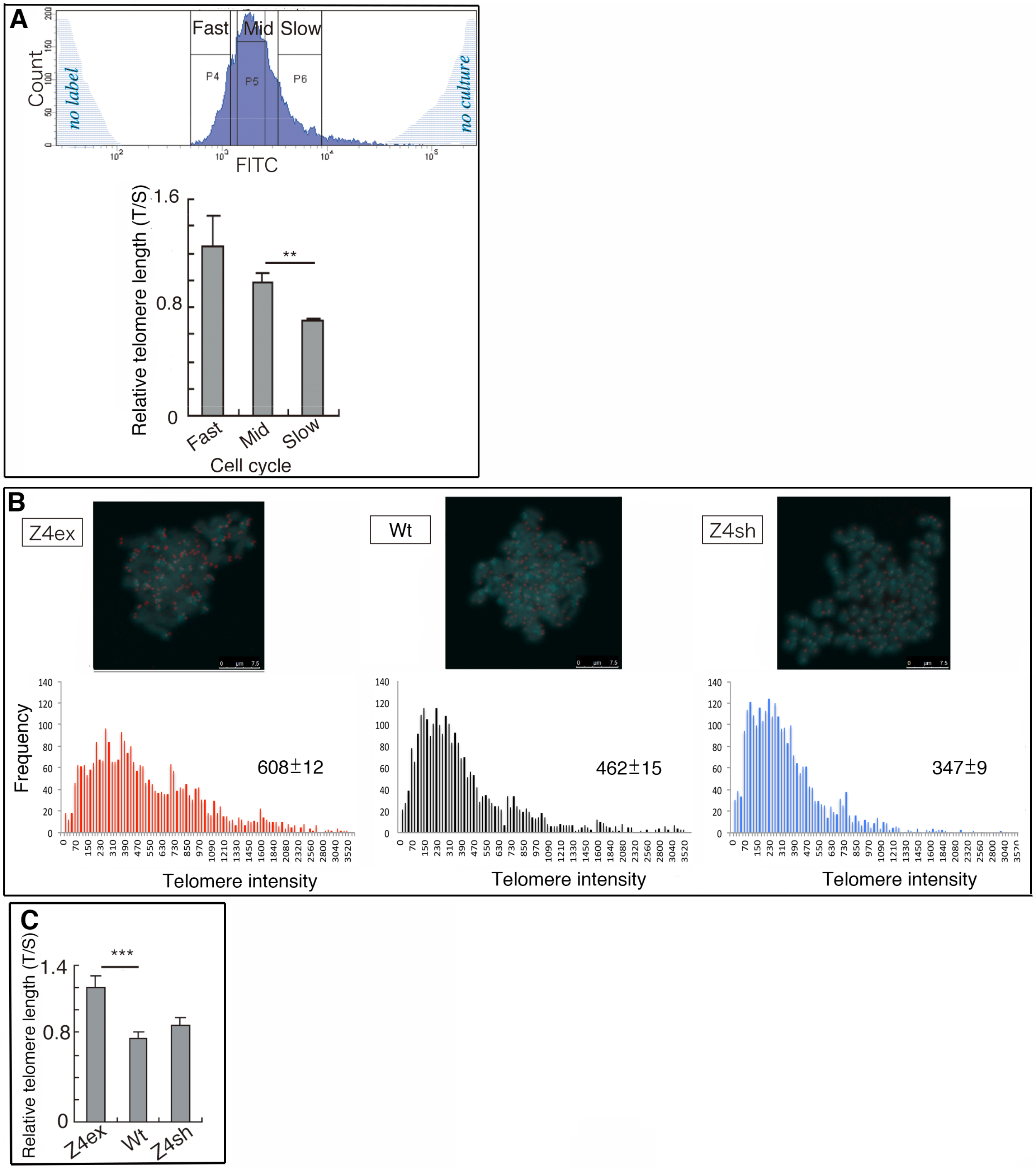

Figure S6, Related to Figures 5 and 6 **Telomere measurement by qPCR and qFISH**

A ESCs labeled with CFSE-dye followed by culture of 48 hours were sorted by FACS into three groups according to the dilution of the dye, which gives cohorts of fast, medium or slow cell cycle (*upper panel*). Telomere length of each cohort was measured by qPCR (n≥3 technical replicates) (*lower panel*). Telomeres were significantly short in the cells with longer cell cycles. **B** Telomere lengths of Z4ex-, wild type- or Z4sh- ESCs measured by qFISH (Bars = 7.5μm; 20 cells each from 2 technical replicates; n=2865, 2803 and 2681 telomeres, respectively). Average telomere intensities ± standard deviations are indicated. **C** Telomere lengths of Z4ex-, wild type- or Z4sh- ESCs measured by qPCR (n≥3 biological replicates) . Unlike qFISH (***B***) and flow-FISH (Figure 6A), the difference between wild-type and Z4-sh-ESCs was not detected, which could be due to the less accuracy of this method for telomere measurement (Gutierrez-Rodrigues et al., 2014). Bar graph represents average with error bars of standard deviation. For statistical analysis, Student's T-test was used. **: $p < 0.005$, ***: $p < 0.001$.

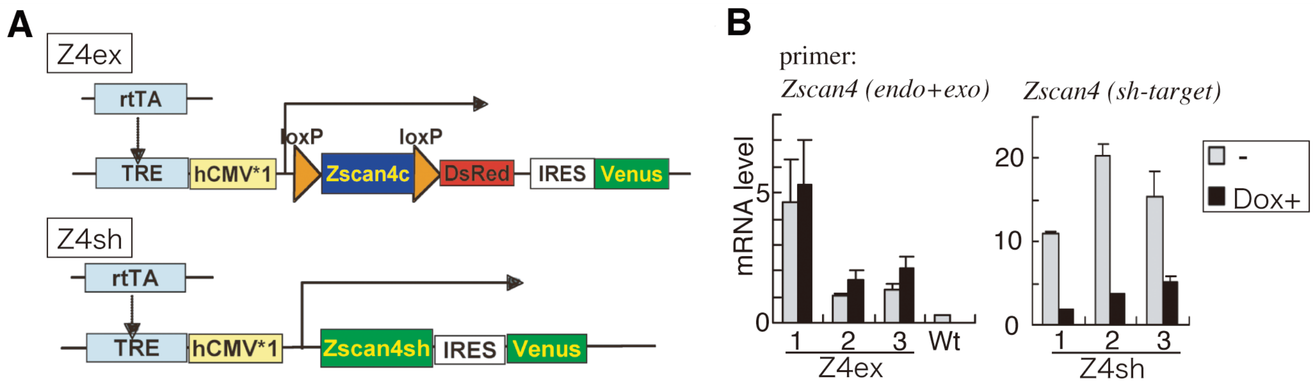

Figure S7, Related to Figure 6   Generation of the Z4ex- and Z4sh- ESCs

A Designs of the *Zscan4c* expression vector in which *Zscan4c* was coded downstream of the Tet-responsible element with a minimal CMV promoter (*upper*) and the Zscan4c knockdown vector in which the same shRNA as previously identified (Zalzman et.al., 2010) was used downstream of the Tet-responsible element with a minimal CMV promoter (*lower*). **B** Tet-inducible expression and knockdown were confirmed by qPCR analysis in Z4ex- and Z4sh- ESCs, using primer pairs that recognize both endogenous and exogenous *Zscan4c* (*endo+exo*) and the target sequence of the *Zscan4c*-shRNA (*sh-target*), respectively. Black bars indicate the Tet-system was ON by the addition of doxycycline (Dox). Bar graph represents average with error bars of standard deviation (n=3 technical replicates).

Note1: The vector design of Z4ex was made to avoid undesired level of over-expression that might kill the cells (Hirata et al., 2012), by flanking *Zscan4c* ORF by loxP so that it could be cleaved by the induction of Cre recombinase after transient expression of *Zscan4c* (Figure S6A). However, unexpectedly, there seemed to be a “leak” in the Tet-system and clones with optimal level of *Zscan4c* that was constitutively expressed even without doxycycline (Dox) were dominantly selected (Figure S6B, *Z4ex* vs *Wt*), which might be a similar case as reported previously (Amano et al., 2013). Driving the Tet-system by the addition of Dox (1 μg/ml) further increased the expression of *Zscan4c* (Figure S6B *Z4ex, Black bars*) but not to a toxic level, as seen by good proliferation and normal morphologies of these clones even in the presence of Dox. Thus to rule out ambiguous “leak” of the Tet-system, we used these clones only in the presence of Dox (i.e. the condition represented by the *Black bars* in Figure S6B *Z4ex*). For controls, instead of Z4ex-ESCs without Dox, wild type ESCs were used. Considering similar leak in Z4sh-ESCs, the Z4sh-ESC lines were used only in the presence of Dox as well (i.e. the condition represented by the *Black bars* in Figure S6B *Z4sh*), with wild type ESCs for controls.

Note2: The knockdown efficiency shown in ***B*** was 83%, 83% and 67% for clones Z4sh#1, #2 and #3, respectively. Clones #1 and #2 were used for the experiments. Although we used the same shRNA sequence as previous study (Zalzman et al., 2010), our knockdown was less effective than Zalzman et al. that showed 96% of reduction. Probably because while we used inducible shRNA, Zalzman et al. stably expressed the shRNA and over-expressed exogenous *Zscan4* during the establishment of the knockdown-clones, which should give more efficient knockdown by continuous expression of the shRNA.

Supplemental Experimental Procedures:

Telomere length measurement by quantitative fluorescent *in situ*-hybridization

Cells were treated with 100 ng/ml of colcemid (Nacalai Tesque, Kyoto, Japan) for 15-19 hours to enrich M-phase cells. Then the cells were trypsinized and incubated in 75mM of KCl at 37 ^{o}C for 20 min, fixed with methanol : acetic acid (3:1), spread on cover glass and dried by sequential wash with 70%, 85% and 96% ethanol. Then the cell-spread was incubated with 30 nM of TelG-Cy3 (Panagene) at 80 ^{o}C for 5 min followed by 2 hours at room temperature. Then the sample was washed sequentially with 70% formamid at room temperature, PBS with 0.1% Tween-20 at 57 ^{o}C and 2xSSC buffer with 0.1% Tween-20 at room temperature. The DNA were counter-stained with Hoechst33342 (Invitrogen).

Images were taken by SP8-confocal microscope (Leica) and the intensities were analyzed using MetaMorph imaging software. To create regions of interest, intensity threshold was set at 5/255 and objects with area size from 10 to 200 pixels were measured.

Telomere length measurement by quantitative PCR

Telomere lengths were measured by qPCR following the method previously described (Callicott and Womack, 2006). Ten ng of genomic DNA was used for 10 ml of reaction mixture of THUNDERBIRD SYBR qPCR Mix. Each reaction was made in triplets. A single copy gene *36B4* was used as an inner control. The telomere signal was normalized to *36B4* to generate a telomere-to-single copy gene (T/S) ratio indicative of relative telomere length. Sequences of primer pairs are as follows:

Telomere (Callicott and Womack, 2006)

forward 5'-CGG TTT GTT TGG GTT TGG GTT TGG GTT TGG GTT TGG GTT-3';

reverse 5'-GGC TTG CCT TAC CCT TAC CCT TAC CCT TAC CCT TAC CCT -3',

36B4 (Callicott and Womack, 2006)

forward 5'-ACT GGT CTA GGA CCC GAG AAG -3';

reverse 5'-TCA ATG GTG CCT CTG GAG ATT -3'.

Supplemental Reference:

Li, X., Zhao, X., Fang, Y., Jiang, X., Duong, T., Fan, C., Huang, C.C., Kain, S.R. (1998). Generation of destabilized green fluorescent protein as a transcription reporter. *J. Biol. Chem.* **273**. 34970-34975.

Supplemental Material:

Source code S1, Related to Figures 2 and 3 **Source code of the program to draw the lineage tree**

This material is provided in a format of a Microsoft Excel sheet (Excel 2003). It includes the hand made program to draw the lineage trees together with the actual input data of Figures 2B and 3C. The source code of the program is embedded as a VBA macro, which can be read and edited by the Microsoft Excel Menu [Tools]-[Macro]-[Visual Basic Editor].
